# Coordination Complexes Built from a Ditopic Triazole-Pyrazole Ligand with Antibacterial and Antifungal Performances

**DOI:** 10.3390/molecules28196801

**Published:** 2023-09-25

**Authors:** Youssef Draoui, Smaail Radi, Mohamed El Massaoudi, Yousra Bahjou, Sabir Ouahhoud, Samira Mamri, Marilena Ferbinteanu, Redouane Benabbes, Mariusz Wolff, Koen Robeyns, Yann Garcia

**Affiliations:** 1 LCAE, Department of Chemistry, Faculty of Science, University Mohamed I, P.O. Box 524, Oujda 60 000, Moroccom.elmassaoudi@ump.ac.ma (M.E.M.);; 2 Institute of Condensed Matter and Nanosciences, Molecular Chemistry, Materials and Catalysis (IMCN/MOST), Université Catholique de Louvain, Place Louis Pasteur 1, 1348 Louvain-la-Neuve, Belgium; koen.robeyns@uclouvain.be; 3 Laboratory of Biochemistry and Biotechnology, Department of Biology, Faculty of Science, University Mohamed I, P.O. Box 524, Oujda 60 000, Morocco; 4 Inorganic Chemistry Department, Faculty of Chemistry, University of Bucharest, Panduri Road, No. 90, 050663 Bucharest, Romania; 5 Institute of Chemical Catalysis, Faculty of Chemistry, Universität Wien, Währinger Straße 38-42, 1090 Wien, Austria; mariusz.jozef.wolff@univie.ac.at

**Keywords:** triazole, pyrazole, microorganisms, bacteria, *Fusarium oxysporum* f. sp. *albedinis*

## Abstract

Four mononuclear complexes (H_3_O){[NiL_3_](ClO_4_)_3_} (**1**), [CoL_3_](ClO_4_)_2_·2H_2_O (**2**), [CdL_2_Cl_2_] (**3**) and [CuL_3_](NO_3_)_2_ (**4**) have been prepared employing a newly synthesized 1,2,4-triazole ligand: 3-(3,5-dimethyl-1*H*-pyrazol-1-yl)-1*H*-1,2,4-triazole (**L**). The structures of the complexes, which crystallized in *P63/m* (**1**), *P-1* (**2**), *P1* (**3**), and *P21/c* (**4**), are reviewed within the context of the cooperative effect of the hydrogen bonding network and counter anions on the supramolecular formations. Moreover, within the framework of biological activity examination, these compounds showed favorable antibacterial performances compared to those of various species of bacteria, including both Gram-positive and Gram-negative strains. Significant antifungal inhibitory activity towards *Fusarium oxysporum* f. sp. *albedinis* fungi was recorded for **3** and **4** over the ligand **L**.

## 1. Introduction

An extensive amount of research has been dedicated to 1,2,4 triazole derivatives due to their diverse applications, for instance, as anticancer agents [1,2], molecular docking and pharmacokinetic [3], anticorrosion activities [4,5], optical properties [6], and antioxidant behaviors [7], to name but a few. For instance, 3-amino-1,2,4-triazole was found to display several biological activities [8], including against microbial infection [9] or as an anti-cancer agent [10], potential radio-sensitizing agent [11], and antiangiogenic agent [10,12,13]. Besides its numerous robust activities, 3-amino-1,2,4-triazole exhibits relevant inherent physico-chemical properties that enhance its bioavailability, chemical stability and general solubility [12,14,15].

Heterocyclic nitrogen compounds occupy a wide portion in the coordination chemistry of transition metal ions. Triazoles are among the best known, and most representative five-membered heterocyclic systems. Due to the presence of at least three nitrogen atoms and an aromatic ring, ligands with a triazole motif are more than qualified for the design of versatile coordination complexes as well as coordination polymers with captivating architectures [16,17]. Those complexes provide many possible properties. For instance, a series of bis (triazole) and carboxylate cobalt coordination polymers were screened for photocatalytic properties [18,19]. Photoluminescence properties were also recently reported by employing zinc and cadmium complexes containing 4-amino-3,5-bis(pyridin-2-yl)-1,2,4-triazole and polynitrile ligands [20]. In addition, many cadmium coordination frameworks were revealed to hold compelling fluorescence properties [21]. Copper triazole-based coordination complexes also demonstrated magnetic and catalytic properties [22] and corrosion inhibition substances [23]. Furthermore, nickel, cobalt, copper and zinc complexes were also proclaimed to bestow variable antibacterial activity against both Gram-positive and Gram-negative bacteria, as well as antifungal activity against *A. niger* and *A. flavus* [24].

Motivated by those applications and properties, triazoles kept sparking the interest and curiosity of our group [25]. Thus, we established a new path towards auspicious novel triazole-based compounds.

In this work, we investigate the synthesis of 3-(3,5-dimethyl-1*H*-pyrazol-1-yl)-1*H*-1,2,4-triazole (**L**), and its characterization via ^1^H NMR, ^13^C NMR, infra-red spectroscopy, UV-visible and high-resolution mass spectrometry. The present ligand afforded four mononuclear complexes, namely (H_3_O){[NiL_3_](ClO_4_)_3_} (**1**), [CoL_3_](ClO_4_)_2_·2H_2_O (**2**), [CdL_2_Cl_2_] (**3**) and [CuL_3_](NO_3_)_2_ (**4**). The complexes were characterized by FT-IR, high-resolution mass spectrometry and single crystal X-ray diffraction. Secondly, the ligand and the coordination complexes were utilized for biological applications. The antibacterial properties of **L** and **1**–**4** were inspected against two Gram-positive bacteria (*Staphylococcus aureus* and *Streptococcus* spp.) and two Gram-negative bacteria (*Escherichia coli* and *Klebsiella* spp.). Their antifungal performance against *Fusarium oxysporum* f. sp. *Albedinis* fungi was also reviewed.

## 2. Result and Discussion

### 2.1. Synthesis of the Ligand (**L**)

**Scheme 1:**
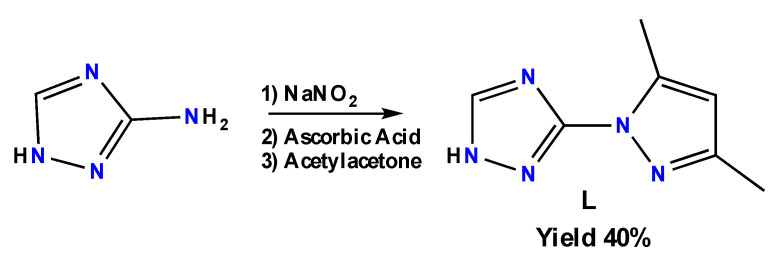
Synthetic route of **L**.

The synthesis route of the ligand **L** (Scheme 1), was adapted from the literature [26]. 3-amino-1,2,4-triazole was reacted with sodium nitrite to form diazonium salt. After one hour, the reduction of the salt was performed using l-ascorbic acid. Acetylacetone was added and after 24 h, 3-(3,5-dimethyl-1*H*-pyrazol-1-yl)-1*H*-1,2,4-triazole (**L**) was obtained with a 40% yield. Ascorbic acid was used instead of SnCl_2_·2H_2_O, which gives impurities and a far worse yield after purification.

### 2.2. Synthesis of the Complexes

**Scheme 2:**
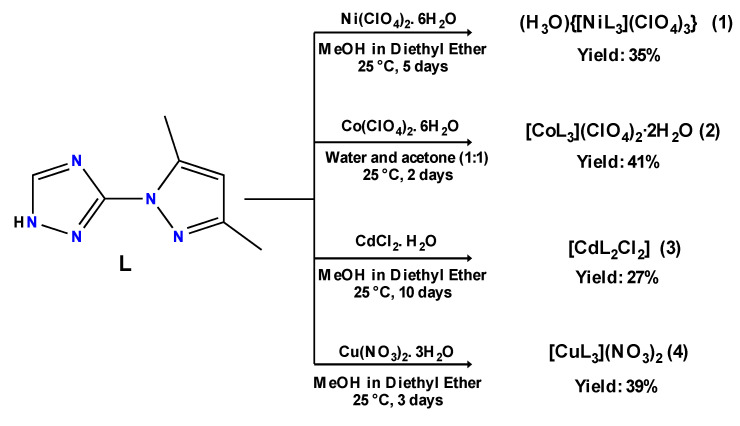
Synthesis of coordination complexes **1**–**4**.

The preparation of coordination complexes **1**–**4** was conducted following the procedure outlined in Scheme 2. Nickel and copper complexes (**1**) and (**4**) were obtained by reacting 3 equiv. of the **L** alongside 1 equiv. of the metal salt Ni(ClO_4_)_2_·6H_2_O and Cu(NO_3_)_2_·3H_2_O, respectively, in methanol. Both single crystals were obtained via vapor-diffusion of diethyl ether in the methanol solution over a couple of days. The cadmium complex (**3**) was also obtained via vapor-diffusion of diethyl ether using only 2 equiv. of **L** in methanol adding some drops of water as CdCl_2_·H_2_O is insoluble in methanol alone.

Lastly, the reaction of 3 equiv. of **L** with 1 equiv. of Co(ClO_4_)_2_·6H_2_O in methanol instantly delivered an orange powder. This powder was then filtered, washed with methanol and dichloromethane, and dried at ambient temperature. Then, to this powder, a mixture of water and acetone (50:50) was added; after that, the resulting mixture was stirred. The corresponding single crystals of (**2**) were grown via slow evaporation.

### 2.3. FT-IR and Diffuse Reflectance Spectroscopies

**Figure 1:**
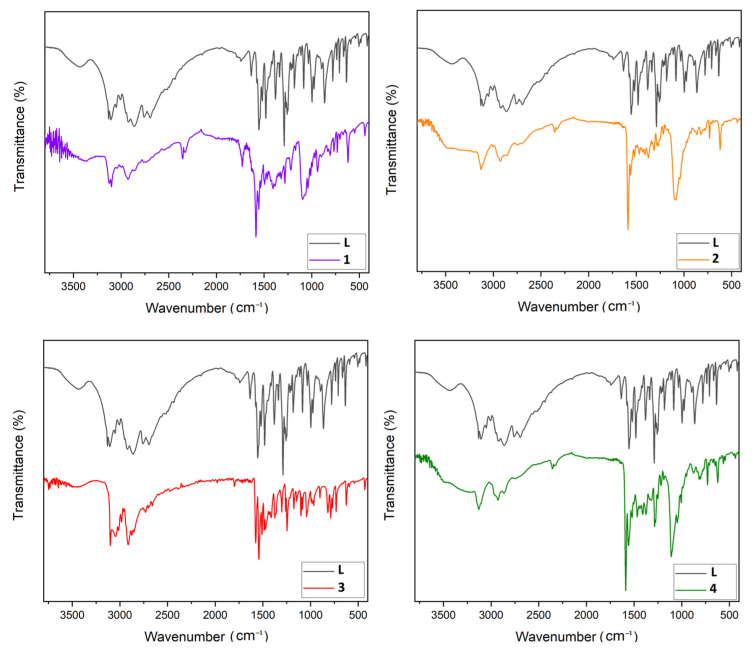
FT-IR spectroscopy comparison plot of **L** with **1**–**4**.

FT-IR spectra comparison of **L**, **1**, **2**, **3**, and **4** are presented in Figure 1. The FT-IR spectrum of 3-(3,5-dimethyl-1*H*-pyrazol-1-yl)-1*H*-1,2,4-triazole revealed characteristic absorption peaks at 2923 and 2859 cm^−1^ due to C-H aromatic vibrations. The N-H stretching vibration peak of the triazole and pyrazole groups was detected at 3131 and 3006 cm^−1^. FT-IR peaks at 1519 and 1481 cm^−1^ were correlated to C=C for aromatic stretching. The vibration peak for –N=N stretching was detected at 1553 cm^−1^. In all these highlighted peaks, complexes (**1**), (**2**), (**3**) and (**4**) witnessed several shifts when compared to the original positioning of the Ligand’s bands. The most representative shift was observed at the 1575 cm^−1^ peak, which is assigned to the C-N imine vibrations of triazole and pyrazole rings. The IR spectra correlation of the ligand to its complexes show that the imine band is shifted up by 10, 17, 3 and 18 cm^−1^ in complexes (**1**), (**2**), (**3**) and (**4**), respectively. This rationalizes the link between the imine of the triazole, as well as the pyrazole, and the transition metal.

Furthermore, we spotted the appearance of new bands at 1098 and 1095 cm^−1^ in the case of the nickel (**1**) and cobalt (**2**) complexes sequentially, which refers to the non-coordinating perchlorate anions, whereas in copper complex (**4**), the signal around 1285 cm^−1^ is specific to the nitrate group [27].

Diffuse reflectance data are shown in Figure S10. **L** shows expected bands assigned to intra-ligand transitions in the UV region, whereas the Cd complex **3** does not absorb in the visible range. On the other hand, complex **1** shows two weak bands around 350 nm and another weak band at λ_max_ ~ 575 nm. The bands were observed in the visible range for the cobalt complex **2** and the copper complex **4** at λ_max_ ~ 500 nm and λ_max_ ~ 670 nm, respectively, as expected for octahedral complexes.

### 2.4. Single Crystal X-ray Measurements

**Figure 2:**
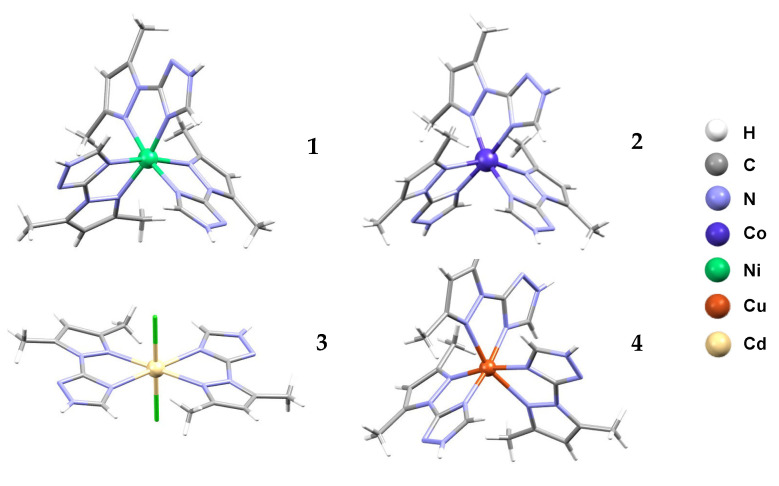
Molecular crystal structures for (H_3_O){[Ni**L**_3_](ClO_4_)_3_} (**1**), [Co**L**_3_](ClO_4_)_2_·2H_2_O (**2**), [Cd**L**_2_Cl_2_] (**3**) and [Cu**L**_3_](NO_3_)_2_ (**4**) with atom coloring scheme. Anions and solvent molecules were omitted for clarity.

Compound **1** crystallizes in the hexagonal system, space group *P* 6_3_/*m* (#176). The crystal structure (Figure 2(1)) shows a distorted octahedral geometry for the nickel ion, which is found on a 3-fold axis (Table S1). The coordination sphere is formed by three symmetry-related bidentate chelating ligands, **L** (Figure S1). Three perchlorate anions are located for every Ni atom, all of them positioned on or close to mirror planes and all heavily disordered. The charge balance is compensated by a hydronium ion, as the nickel ion is found on a 3-fold axis. The coordination complexes and the anions are arranged in discrete layers (Figure S2), held together by hydrogen bonds between the triazole N-H and a perchlorate anion. Parallel-displaced π-π stacking interactions are observed between pyrazole rings from a neighboring complex molecule that are linked by an inversion center (Figure S3).

Compound **2** crystallizes with two non-coordinated water molecules, in the triclinic system, space group *P*-1 (#2). The molecular structure of the complex (Figure 2(2)) is similar to the that of the Ni analogue (Figure S4), showing a tris-chelate complex of cobalt ion with the ligand **L** and two charge-compensating perchlorate ions (Figure S5). No hydronium ion was found.

Compound **3** crystallizes in the triclinic system, space group *P*-1 (#2). The crystal structure shows a mononuclear cadmium complex (Figure 2(3)). The Cd atom is located on an inversion center (Figure S6), with two chelating ligands **L** in equatorial positions and two chloride anions in the axial positions (Figure S7).

Compound **4** crystallizes in the monoclinic system, space group *P* 2_1_/*c* (#14). The metal complex, Cu(II) (Figure 2(4)), is similar to compounds **1** and **2** (Figure S8), as the anions are nitrates, but no lattice water molecules are present (Figure S9).

In all of the structures reported here, the ligand, **L**, is coordinated in a bidentate mode by one nitrogen donor atom from the triazole (trz) ring and another nitrogen donor atom from the pyrazole (pz) fragment. The bond lengths M-N(trz) in compounds **1**, **2** and **4** are shorter than for M-N(pz) one (Table S1) and all vary with the ionic metal volume. The octahedral geometry in **4** is more distorted than that in the similar compound **2**, due to the more pronounced Jahn–Teller effect. The ratio between the M-N(trz) and M-N(pz) bond lengths in the compound **3** is inverse, maybe due to the presence of the chloride ions in the apical position or to the absence of the sterically hindrance from tris chelates compounds **1**, **2** and **4**.

The distortion parameter Σ for **1** is 74.99°, which is lower than the value reported [Ni(L’)_2_](ClO_4_)_2_ with L’ = diethyl 1,10-(pyridine-2,6-diyl) bis(5-methyl-1*H*-pyrazole-3-carboxylate) [28]. On the other hand, complexes **2**, **3** and **4** have rather similar values (Table 1). We recall that Σ measures local angular distortions of the octahedral donor set, where α_i_ are the 12 *cis*-N–M–N angles at the metal center [28] following Equation (1):

(1) $$\Sigma =\sum_{i=1}^{12}⎪90-\alpha i⎪$$

Two parameters are considered, Σ and Θ, which specify the degree of trigonal distortion of the coordination geometry from an octahedron to a trigonal prism (Equation (2)):

(2) $$\Theta =\sum_{j=1}^{24}⎪60-\beta j⎪$$

where β_j_ is the 24 unique torsion angles between adjacent N donors on opposite triangular faces of the octahedron, measured along their common (pseudo)-three-fold axis [29].

### 2.5. Antibacterial Activities

**Figure 3:**
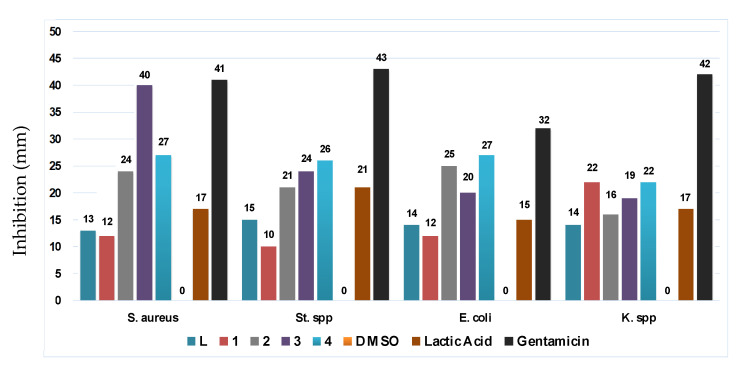
Antibacterial activities recap plot of **L** and the coordination complexes **1**–**4**.

The ligand **L** and the coordination complexes **1**–**4** were evaluated against *Staphylococcus aureus* and *Streptococcus* spp. as Gram-positive bacteria, as well as *Escherichia coli* and *Klebsiella* spp. as Gram-negative bacteria. The results are shown in Table 2 and Figure 3.

Complexes **1** and **4** revealed a notable improvement against *Klebsiella* spp. Besides complex **2**, **3** and **4** also show a major increase in terms of inhibition as regards *Staphylococcus aureus*, *Streptococcus* spp. and *Escherichia coli* that even exceeds inhibition by lactic acid. The cadmium complex [CdL_2_Cl_2_] (**3**) exhibits an outstanding result of 40 mm for the inhibition of Staphylococcus aureus, which is significantly higher than that of lactic acid while being as good as Gentamicin. This value is found to be even better compared to those found in the literature on cadmium complexes [30,31]. The second remarkable value is given by complex [CuL_3_](NO_3_)_2_ (**4**) with 27 mm. The best performance against *Streptococcus* spp. was recorded for **4** with 26 mm followed by **3** with a 24 mm. Furthermore, **2** and **4** earned the top spot vs. *Escherichia coli* bacteria, with 25 mm and 27 mm, respectively. Lastly, the best value for the *Klebsiella* spp. was reached by **1** and **4** with a similar 22 mm inhibition zone. The results mentioned above are all significantly better than lactic acid’s performance, in addition to remaining globally competitive when compared to literature values of triazole derivatives. It has been reported, for instance, that 1,2,4-triazole ligands as well as their coordination complexes accomplished considerably lower results even at 10 mg/L concentration against *Escherichia coli* [32]. Additionally, four triazole copper complexes revealed lower inhibition vs. *Staphylococcus aureus* and *Escherichia coli* compared to the copper complex **4** even at the same concentration range [24]. Our results also performed considerably better compared to other copper-, cadmium- and zinc-Schiff-based coordination complexes despite their higher concentration (5 mg/mL) [33].

### 2.6. Antifungal Activities

**Figure 4:**
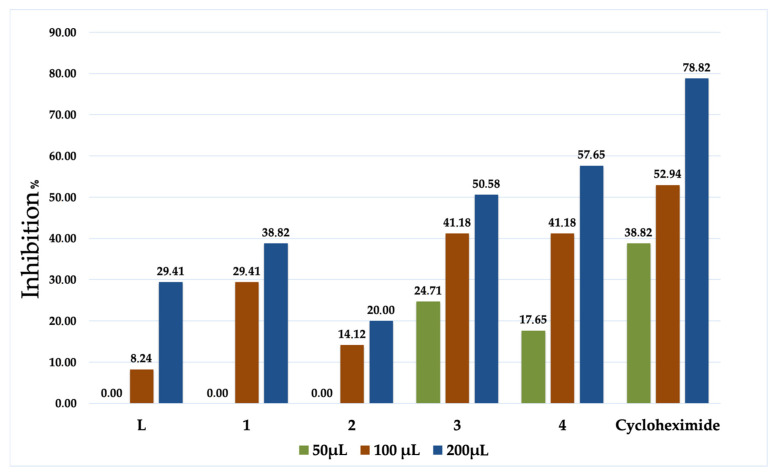
Antifungal activities of **L** and the coordination complexes **1**–**4**.

Date palm (*Phoenix dactylifera* L.) is an essential food source and has been a commercial cultivation in North Africa for a long time. “Bayoud”, a vascular wilt disease triggered by the soilborne fungal pathogen *Fusarium oxysporum* f. sp. *albedinis* (FOA), has become a genuine menace to date production in date-palm-growing regions in Morocco [34]. In this regard, our compounds were also investigated against FOA (Table 3 and Figure 4).

All of the evaluated samples indicate an anti-Fusarium attitude, as shown in Table 3. Complexes **3** and **4** illustrate exceptional inhibition records even at low concentrations, both of which performed considerably well at 100 µL, and as well as Cycloheximide; in fact, the statistical analysis revealed that the difference in inhibition % is insignificant when compared to the positive control. This is mainly due to the lower concentration of **3** and **4** applied. These performances are mainly due to the effect of the transition metals, which boost the lipophilicity of the coordination complexes, therefore facilitating their permeability through the lipid bilayer of membranes [35]. In addition, these complexes hold a 2:1 and 3:1 stoichiometry ratio; hence, their size is two to three times that of the ligand [36]. To further approve this fruition, **3** and **4** were tested three times to determine the required volume to reach a 50% inhibition; the results were 89.9 and 66.1 µmol/L, respectively. Judging by all the collected information with these anti-Fusarium experiments, we can therefore acknowledge the efficiency of the product used especially in the case of **3** and **4.** These upshots set the abovementioned compounds drastically beyond a large number of specimens reported in the literature [37,38,39].

## 3. Experimental Section

### 3.1. Material and Instrumentation

All solvents and chemicals, obtained from usual commercial sources, were of analytical grade and used without further purification. ^1^H and ^13^C NMR spectra were obtained from a Bruker AC 300 spectrometer. High-resolution electrospray ionization mass spectra (HR-ESI-MS) were recorded on a *maXis* QTOF-MS instrument (Bruker Daltonics GmbH, Bremen, Germany). FT-IR spectra were recorded on potassium bromide discs using a Perkin Elmer 1310 spectrometer (PerkinElmer, Waltham, MA, USA). Melting points were measured using a Koffler bench. UV-visible spectra were recorded using a Shimadzu 3600 plus spectrometer (Shimadzu, Kyoto, Japan) equipped with Harrick praying mantis modulus, which enables a direct analysis of the powders in reflectance mode.

### 3.2. X-ray Crystallography

Crystal data for **1**, **2** and **4** have been collected on a MAR345 Image plate detector using a monochromated (montel optics) microfocus Mo-Kα radiation (*λ* = 0.71073 Å) source (Incoatec IµS). Data integration and reduction were performed using the CrysAlis^PRO^ V 1.171. 37.35 crystallographic software package and the implemented absorption correction was used [40]. Crystal data for **3** have been collected on a Rigaku R-AXIS RAPID II diffractometer (Rigaku, Tokyo, Japan) using graphite monochromated Mo-Kα radiation (λ = 0.71075 Å) and the ω-ϕ scan technique. All structures were solved via dual space direct methods (SHELXT) and refined using SHELXL2018/3 [41]. Nonhydrogen atoms were refined anisotropically with hydrogens placed in calculated positions and refined in riding mode. The structure of **1** contains voids (a total of 164Å^3^ or 4.2% of the unit cell volume, distributed over 2 equivalent pockets, in which a total of 19e^−^ were found), for which the disordered electron density was treated using the squeeze algorithm in PLATON [42]. In **1** and **2**, almost all ClO_4_^–^ anions were found disordered and refined over two sites; isotropic and rigid bond restraints were used to refine the ClO_4_^–^ anions.

Details of the crystal parameters, data collection and refinement for the compounds are listed in Table S2. A summary of selected bond lengths [Å] and angles [°] are given in Table S1 (ESI). CCDC 2206409-2206412 contains the supplementary crystallographic data for this paper. These data can be obtained free of charge from The Cambridge Crystallographic Data Centre via www.ccdc.cam.ac.uk/structures, accessed on 20 September 2023.

### 3.3. Synthesis Section

Synthesis of 3-(3,5-dimethyl-1*H*-pyrazol-1-yl)-1*H*-1,2,4-triazole (**L**):

A solution of sodium nitrite (5.3 g, 76.82 mmol, 1.2 equiv.) in water (10 mL) was added dropwise to a stirred solution of 1*H*-1,2,4-triazol-3-amine (5.38 g, 64.01 mmol, 1 equiv.) in 36 mL of 12 M hydrochloric acid at 0 °C. After stirring for 1 h at the same temperature, a solution of ascorbic acid (11.28 g, 64.04 mmol, 1 equiv.) in cold water (54 mL) was added drop wise at 0 °C. After 10 min of stirring, a solution of acetylacetone (6.4 g, 64.04 mmol, 1 equiv.) with a few drops of ethanol was added dropwise to the reaction mixture and left under stirring at room temperature for one day. The reaction mixture was then neutralized with sodium carbonate to pH = 7; the aqueous phase was extracted 3 times with chloroform (20 mL), dried with magnesium sulphate, and then evaporated to dryness. Diethyl ether (30 mL) was added to the resulting mixture and left at 0 °C overnight. A white powder was obtained after filtration. M.p.: 134 °C. Yield: 40% (4.1 g). FT-IR: 3130(w), 3006(w), 2923(w), 2859(w), 1575(m), 1553(s), 1520(m), 1481(m). ^1^H-NMR (Chloroform) *δ* ppm: 2.61 (s, 3H, CH_3_-C=N), 2.74 (s, 3H, CH_3_-C-N), 6.76 (s, 1H, C=CH-C), 8.37 (s, 1H, N-CH=N). ^13^C-NMR (Chloroform) *δ ppm*: 17.0 (CH_3_-C=N-N-trz), 17.0 (CH_3_,C-N-trz), 110.7 (C=CH-C), 146.6 (N-C=CH) 155.4 (N-C-N), 155.4 (N=C-CH), 164.8 (HN-CH=N). ESI (+) *m*/*z* found = 164.0938, *m*/*z* calcd. for (M+H)^+^ = 164.09307; *m*/*z* found = 186.0755, *m*/*z* calcd. for (M+Na)^+^ = 186.07502.

Synthesis of (H_3_O){[NiL_3_](ClO_4_)_3_} (**1**):

**L** (49 mg, 0.3 mmol, 3 equiv.) was dissolved in methanol (3 mL). Ni(ClO_4_)_2_·6H_2_O (36.5 mg, 0.1 mmol, 1 equiv.) was dissolved in methanol (3 mL) and added to the previous solution of **L** and stirred for 10 min at room temperature. The resulting violet liquid mixture was allowed to vapor-diffuse with diethyl ether (10 mL) at room temperature. Violet single crystals were obtained after 5 days. Yield 35% (30 mg). FT-IR: 3126(w), 2929(w), 1585(s), 1575(m), 1098(m), 830(w), 618(w). ESI (+): *m*/*z* found = 383.0991, *m*/*z* calcd. for [Ni_2_(C_7_H_9_N_5_)_2_(C_7_H_8_N_5_)_2_]^2+^ = 383.09856; *m*/*z* found = 493.6006, *m*/*z* calcd. for [Ni_3_(C_7_H_8_N_5_)_4_(C_7_H_9_N_5_)]^2+^ = 493.59941; *m*/*z* found = 602.1052, *m*/*z* calcd. for [Ni_2_(C_7_H_8_N_5_)_3_]^+^ = 602.10514; *m*/*z* found = 767.1880, *m*/*z* calcd. for [Ni_4_(C_7_H_8_N_5_)_6_(C_7_H_9_N_5_)_2_]^2+^ = 767.17959; ESI-MS (–): *m*/*z* found = 519.8618, *m*/*z* calcd. for [Ni(C_7_H_9_N_5_)(ClO_4_)_3_]^–^ = 519.86397; *m*/*z* found = 682.9492, *m*/*z* calcd. for [Ni(C_7_H_9_N_5_)_2_(ClO_4_)_3_]^–^ = 682.94984; *m*/*z* found = 846.0359, *m*/*z* calcd. for [Ni(C_7_H_9_N_5_)_3_(ClO_4_)_3_]^–^ = 846.03576.

Synthesis of [CoL_3_](ClO_4_)_2_·2H_2_O (**2**):

**L** (49 mg, 0.3 mmol, 3 equiv.) was dissolved in methanol (3 mL). Co(ClO_4_)_2_·6H_2_O (36.5 mg, 0.1 mmol, 1 equiv.) was dissolved in methanol (3 mL) and added to the previous solution of **L**, and stirred for 20 min at room temperature. The resulting powder was filtered off, washed with methanol and dichloromethane, dried at room temperature and then dissolved in a 4 mL mixture of (50:50) water and acetone (2 mL each). The resulting orange liquid mixture was stirred for 10 min, then left at room temperature for slow evaporation. Orange single crystals were obtained after 2 days. Yield 41% (32 mg). FT-IR: 3125(w), 2939(w), 1592(s), 1557(m), 1095(m), 865(w), 620(w). ESI-MS (+) *m*/*z* found = 384.0960; *m*/*z* calcd. for ([Co(C_7_H_8_N_5_)_2_]+H)^+^ = 384.09641. ESI-MS (–): *m*/*z* found = 518.6844, *m*/*z* calcd. for [Co(C_7_H_9_N_5_)(ClO_4_)_3_]^–^ = 518.86507; *m*/*z* found = 683.9465, *m*/*z* calcd. for [Co(C_7_H_9_N_5_)_2_(ClO_4_)_3_]^–^ = 683.94823.

Synthesis of [CdL_2_Cl_2_] (**3**):

**L** (32,7 mg, 0.2 mmol, 2 equiv.) was dissolved in methanol (3 mL). CdCl_2_·H_2_O (20.1 mg, 0.1 mmol, 1 equiv.) was dissolved in methanol (3 mL) with five drops of water, and then added to the previous solution of **L**, and stirred for 10 min at room temperature. The resulting clear liquid mixture was allowed to vapor-diffuse with diethyl ether (10 mL) at room temperature. Colorless single crystals were obtained after 10 days. Yield 27% (14 mg). FT-IR: 3102(w), 2912(w), 1578(s), 1543(m), 823(w), 630(w). ^1^H NMR (600 MHz, DMSO-d_6_) *δ* ppm: 14.33 (s, 1H, N–H), 8.67 (s, 1H, N–CH=N), 6.11 (s, 1H, C=CH–C), 4.09 (CH_3_OH), 3.32 (H_2_O), 3.17 (CH_3_OH), 2.50 (DMSO-*d*_6_), 2.40 (s, 3H, CH_3_–C–N), 2.21 (s, 3H, CH_3_–C=N). ^13^C-NMR (DMSO-d_6_) *δ ppm*: 12.3 (CH_3_-C=N-N-trz), 13.2 (CH_3_,C-N-trz), 39.5 (DMSO-*d*_6_), 48.6 (CH_3_OH), 107.7 (C=CH-C), 140.9 (N-C=CH), 144.0 (N-C-N), 149.2 (N=C-CH), 156.8 (HN-CH=N). ESI-MS (+): *m*/*z* found = 311.9570, *m*/*z* calcd. for [Cd(C_7_H_9_N_5_)Cl]^+^ = 311.95685; *m*/*z* found = 475.0425, *m*/*z* calcd. for [Cd(C_7_H_9_N_5_)_2_Cl]^+^ = 475.04280.

Synthesis of [CuL_3_](NO_3_)_2_ (**4**):

**L** (49 mg, 0.3 mmol, 3 equiv.) was dissolved in methanol (3 mL). Cu(NO_3_)_2_·3H_2_O (24.1 mg, 0.1 mmol, 1 equiv.) was dissolved in methanol (3 mL) and then added to the previous solution of **L**, as well as stirred for 10 min at room temperature. The resulting green liquid mixture was allowed to vapor-diffuse with diethyl ether (10 mL) at room temperature. Green single crystals were obtained after 3 days. Yield 39% (26 mg). FT-IR: 3127(w), 2932(w), 1593(s), 1575(m), 1285(m), 875(w), 622(w). ESI-MS (+) *m*/*z* found = 419.5499, *m*/*z* calcd. for [Cu_3_(C_7_H_8_N_5_)_4_]^2+^ = 419.54904; *m*/*z* found = 612.0940, *m*/*z* calcd. for [Cu_2_(C_7_H_8_N_5_)_3_]^+^ = 612.09256.

### 3.4. Antibacterial Activities

#### 3.4.1. Bacterial Strains

The bacteria *Staphylococcus aureus* (*S. aureus*), *Streptococcus* spp. (*St.* spp.), *Escherichia coli* (*E. coli*), and *Klebsiella* spp. (*K.* spp.) that were used to test the antibacterial activity were clinically isolated and identified in the microbiology lab of the University Hospital Centre Mohammed VI of Oujda (Morocco). The European Committee on Antimicrobial Susceptibility Testing’s (EUCAST) guidelines were used to determine their antibiotic sensitivity. The strains chosen are typical of multi-resistant bacteria that commonly cause human infections.

#### 3.4.2. Antibacterial Activity Evaluation

Antibacterial activities were determined using a slightly modified disk diffusion technique on agar [43,44]. In sterile Petri dishes (Ø 90 mm), 15 mL of Mueller–Hinton agar (45 °C) were poured.

Each organism was taken from a previous culture (Mueller–Hinton agar medium) and suspended in a normal saline solution individually and measured for transmittance (T) of 75–77% at *λ* = 530 nm, which equals 10^6^ CFU/mL. In a Petri dish, each bacterial suspension was evenly dispersed on a firm growth substrate.

In total, 4 mg of each compound (**L** and **1**–**4**), as well as the positive controls were dissolved in DMSO (1 mL). Holes measuring 6 mm were drilled into the agar after the plates had been aseptically dried using a sterile Pasteur pipette. In the holes, 100 µL of the previously prepared solution containing each component was poured. Then, the plates were incubated in an oven for 24 h at 37 °C.

Since DMSO has no antibacterial properties, it was used as a negative control. Lactic acid, on the other hand, was used as a positive control due to its decent inhibitory activity against various bacteria types, while the antibiotic gentamycin was designated as the ideal positive control. Positive antimicrobial activity was determined using the diameters of the inhibition zones obtained around the holes and compared to the solvent as a control. For each bacterial strain, we performed an experiment with three repetitions, i.e., three plates ran on the same days with the same bacterial cultures.

### 3.5. Antifungal Activities

#### 3.5.1. Preparation of the Substances

The stock solution was prepared by dissolving 4 mg of each compound (**L** and **1**–**4**) in DMSO (1 mL).

#### 3.5.2. Preparation of the Fungus

First, the FOA (*Fusarium oxysporum* f. sp. *albedinis*) was isolated from xylem tissues exhibiting characteristic “Bayoud” symptoms on Boufegousgharas date palm in Figuig, Morocco. Vascular tissue fragments were cut off and aseptically put in PDA medium before being incubated at 28 °C. *Fusarium oxysporum* isolates were identified based on their morphological characteristics [45,46]. On PDA medium at 28 °C, one *Fusarium oxysporum* monoconidial was isolated.

#### 3.5.3. Antifungal Activities Protocol

Potato Dextrose Agar (PDA) (39 g) was mixed with distilled water (1 L), heated under agitation until ebullition was reached in order to homogenize the medium. The mixture was then sterilized at 120 °C for 20 min. After cooling, three sterile tubes were filled with a volume of 50 µL, 100 µL, and 200 µL of each stock solution containing the compounds previously prepared, then completed with liquid PDA to a volume of 15 mL, spread over 8.5 cm diameter Petri dishes, and left at room temperature until solidification [47]. Using sterile Pasteur pipette, a 6mm disk of the grown FOA was then transplanted in the center of each Petri dish. The prepared Petri dishes were incubated for 5 days at 28 °C. The results are expressed as a percentage inhibition by measuring the diameter of the FOA in comparison to the negative control (200 µL) containing only PDA, FOA, and DMSO [48], while the cycloheximide was used as positive control. The experiment was performed in triplicate, i.e., three plates ran on the same days with the same FOA culture.

(3) $$\%\;\mathrm{of}\;\mathrm{inhibition}=\frac{D_{0}-D_{x}}{D_{0}}\times 100$$

D_0_ diameter (cm) of FOA in the control;
D_x_: diameter (cm) of FOA in the test.

### 3.6. Statistical Analysis

With n = 3, the data were presented as mean ± SEM. One-way ANOVA was used to carry out statical analysis using the GraphPad Prism 8.0 program. With significant levels of *p* < 0.05, Sidak’s multiple comparison test was used to examine the differences between the means of various groups.

## 4. Conclusions

In conclusion, to overcome the solubility problem with our targeted 1,2,4-triazole molecule, we have employed a straightforward method using acid. This ligand effortlessly granted us four hexacoordinated mononuclear complexes, one of them (**3**) welcoming two chloride atoms in its coordination sphere instead of six nitrogen atoms. The ligand, as well as the coordination complexes, performed well in terms of antifungal activities, especially **3** and **4**, even at low concentrations, compared to the results found in the literature.

Regarding the antibacterial activities, compounds **L** and **1**–**4** displayed high performance compared to that of lactic acid as a positive control even at significantly lower concentrations in all tested bacteria. For instance, complex **1** exhibits a 22 mm inhibition vs. *Klebsiella* spp., which is superior compared to that of lactic acid even at a concentration 10 times lower. The outstanding results regarding antibacterial activities for **3** over *Staphylococcus aureus* despite the low concentration used (7.85 mmol/L) encourage us to continue to explore more possibilities of adjacent ligands in future work.

## Figures and Tables

**Table 1:** Distortion parameters Σ and Θ of **1**–**4**.

Complex	1	2	3	4
Σ°	74.99	100.72	91.88	93.36
Θ°	237.71	316.03	357.02	283.03

**Table 2:** Antibacterial activities of **L** and the complexes **1**–**4** against four strains (inhibition zones in mm).

Compound	Concentration (mmol/L)	Bacteria			
		*S. aureus* [p]	(*St.* spp.) [p]	*E. coli* [n]	(*K.* spp.) [n]
DMSO	Solvent	0 ^#,^*	0 ^#,^*	0 ^#,^*	0 ^#,^*
**L**	24.51	13 ± 0.38 ^#^	15 ± 0.64 ^#,^*	14 ± 0.51 ^#^	14 ± 1.02 ^#^
**1**	4.62	12 ± 0.25 ^#,^*	10 ± 0.25 ^#,^*	12 ± 0.38 ^#^	22 ± 0.64 ^#,^*
**2**	5.11	24 ± 0.51 ^#,^*	21 ± 0.64 ^#^	25 ± 0.25^#,^*	16 ± 0.89 ^#,^*
**3**	7.85	40 ± 0.25 *	24 ± 0.64 ^#,^*	20 ± 0.38 ^#,^*	19 ± 1.15 ^#,^*
**4**	5.91	27 ± 0.19 ^#,^*	26 ± 0.25 ^#,^*	27 ± 0.25 ^#,^*	22 ± 0.64 ^#,^*
Lactic Acid	44.40	17 ± 0.70 ^#^	21 ± 0.76 ^#^	15 ± 0.64 ^#^	17 ± 0.25 ^#^
Gentamicin	8.38	41 ± 0.35 *	43 ± 0.38 *	32 ± 0.38 *	42 ± 1.02 *

[p]: Gram-positive; [n]: Gram-negative. ^#^ *p* < 0.05 compared with Gentamicin; * *p* < 0.05 compared with lactic acid.

**Table 3:** Antifungal activities of **L** and the complexes **1**–**4**.

Compound	Sample Volumes (µL)	Concentration (µmol/L)	D_x_ (mm)	Inhibition (%)
**L**	50	81.7	85 ± 0.51	0 *
	100	163.4	78 ± 0.62	8.24 *
	200	326.8	60 ± 0.25	29.41 ^#,^*
**1**	50	15.4	85 ± 0.33	0 *
	100	30.8	60 ± 0.21	29.41 ^#,^*
	200	61.6	52 ± 0.72	38.82 ^#,^*
**2**	50	17	85 ± 1.09	0 *
	100	34	73 ± 0.82	14.12 ^#,^*
	200	68.1	68 ± 0.17	20.00 ^#,^*
**3**	50	26.2	64 ± 0.42	24.71 ^#,^*
	100	52.3	50 ± 0.11	41.18 ^#^
	200	104.6	42 ± 1.11	50.58 ^#,^*
**4**	50	19.7	70 ± 0.66	17.65 ^#,^*
	100	39.3	50 ± 0.41	41.18 ^#^
	200	78.8	36 ± 0.29	57.65 ^#,^*
Cycloheximide	50	47.4	52 ± 0.15	38.82 ^#^
	100	94.78	40 ± 0.15	52.94 ^#^
	200	189.6	18 ± 0.1	78.82 ^#^

^#^ *p* < 0.05 compared with negative control; * *p* < 0.05 compared with Cycloheximide.

## Data Availability

Upon reasonable request from the authors.

## Supplementary Materials

The following supporting information can be downloaded at: https://www.mdpi.com/article/10.3390/molecules28196801/s1, Tables S1–S2 and Figures S1–S9: Crystallographic information for **1**–**4**; Figure S10: Diffuse reflectance spectroscopy of **L** and **1**–**4**; ^1^H NMR, ^13^C NMR and HRMS of **L**. HRMS for **1**–**4**.

## Sample Availability

Samples of the compounds **L**, **1**, **2**, **3**, and **4** are available from Y.D.
