# Supplementary material for: Coordination Complexes Built from a Ditopic Triazole-Pyrazole Ligand with Antibacterial and Antifungal Performances
Source: Molecules. 2023 Sep 25;28(19):6801. doi: 10.3390/molecules28196801 (PMC10574422; doi:10.3390/molecules28196801)
Supplement: Supplementary file 1 [file molecules-28-06801-s001.zip › molecules-2625270-supplementary.pdf]

Electronic Supplementary Information (ESI)

Coordination Complexes Built from a Ditopic Triazole-Pyrazole Ligand with Antibacterial and Antifungal Performances

Youssef Draoui [1], Smaail Radi [1,*], Yousra Bahjou [1,2], Mohamed El Massaoudi [1], Sabir Ouahhoud [3], Samira Mamri [3], Marilena Ferbinteanu [4], Redouane Benabbes [3], Mariusz Wolff [5], Koen Robeyns [2], Yann Garcia [2,*]

[1] LCAE, Department of Chemistry, Faculty of Science, University Mohamed I , P.O. Box 524, Oujda 60 000, Morocco.

[2] Institute of Condensed Matter and Nanosciences, Molecular Chemistry, Materials and Catalysis (IMCN/MOST), Place Louis Pasteur 1, Université catholique de Louvain, Belgium

[3] Laboratory of Biochemistry and Biotechnology, Department of Biology, Faculty of Science, University Mohamed I , P.O. Box 524, Oujda 60 000, Morocco.

[4] Inorganic Chemistry Department, Faculty of Chemistry, University of Bucharest, Panduri Road, no. 90, Bucharest 050663, Romania

[5] Institut für Chemische Katalyse, Fakultät für Chemie, Universität Wien, Währinger Straße 38-42, 1090 Wien, Austria

Correspondence : yann.garcia@uclouvain.be ; s.radi@ump.ac.ma

Contents:

Table S1. Selected bond lengths [Å] and angles [°] for **1-4**……4

Table S2. Summary of crystallographic data and refinement for **1-4**……6

Figure S1. Molecular crystal structures for $(H_3O)\{[Ni\mathbf{L}_3](ClO_4)_3\}$ (**1**), displacement ellipsoids drawn at the 50% probability level and atom numbering scheme. The ClO_4^- anion and H_3O cation were omitted for clarity……7

Figure S2. Packing details for **1** along the *a* axis. Anions and solvent molecules are represented with spacefill style……..8
Figure S3. Packing details for **1** showing the π-π stacking interaction between pyrazole rings from neighboring complex molecules………9
Figure S4. Molecular crystal structures for $[CoL_3](ClO_4)_2 \cdot 2H_2O$ (**2**), displacement ellipsoids drawn at the 50% probability level and atom numbering scheme. The ClO_4^- anions and H_2O molecule were omitted for clarity. Atom numbering for the other ligands is incremented by 20……..10
Figure S5. Packing details for **2** along the *a* axis. Anions and solvent molecules are represented with spacefill style. Hydrogen atoms are omitted for clarity…………………………………………………………………………………………………..11
Figure S6. Molecular crystal structures for $[CdL_2Cl_2]$ (**3**), displacement ellipsoids drawn at the 50% probability level and atom numbering scheme..…………………………………………………………………………………………………......12
Figure S7. Packing details for **3** along the *a* axis showing hydrogen bonding……..…………………………………13
Figure S8. Molecular crystal structures for $[CuL_3](NO_3)_2$ (**4**), displacement ellipsoids drawn at the 50% probability level and atom numbering scheme. Anions were omitted for clarity. Atom numbering for the other ligands is incremented by 20..…….…14
Figure S9. Packing details for **4** along the *a* axis. Anions are represented with spacefill style………...…..15
Figure S10. Diffuse reflectance spectroscopy comparison plot of **L** with **1- 4**……………………………………..16
^{1}H NMR L…………………………………………………………………………………………...……17
^{13}C NMR L ………………………………………………………………………………..………18
1H NMR spectrum of complex 3……………………………………………………………………….20
^{13}C NMR spectrum of complex 3……………………………………………………………………21

HRMS L……………………………………………………………………………………………………21
HRMS 1 ……………………………………………………………………………………………………23
HRMS 2……………………………………………………………………………………………………28
HRMS 3……………………………………………………………………………………………………32
HRMS 4……………………………………………………………………………………………………36

Table S1. Selected bond lengths [Å] and angles [°] for **1-4.**

Compound	Bond lengths, Å	Bond angles, °
**1**	Ni1-N10* 2.073(3)<br>Ni1-N10* 2.073(3)<br>Ni1-N10* 2.073(3)<br>Ni1-N2** 2.146(4)<br>Ni1-N2** 2.146(4)<br>Ni1-N2** 2.146(4)	N10-Ni1-N10 93.79(13)<br>N10-Ni1-N2 169.45(14)<br>N10-Ni1-N2 77.94(14)<br>N10-Ni1-N2 93.30(13)<br>N10-Ni1-N2 169.44(14)<br>N10-Ni1-N2 77.94(14)<br>N2-Ni1-N2 95.83(13)
**2**	Co1-N30* 2.132(3)<br>Co1-N10* 2.135(3)<br>Co1-N50* 2.144(2)<br>Co1-N22** 2.161(3)<br>Co1-N42** 2.176(2)<br>Co1-N2** 2.179(3)	N30-Co1-N10 174.69(10)<br>N30-Co1-N22 76.49(9)<br>N10-Co1-N22 98.77(10)<br>N30-Co1-N42 95.16(10)<br>N50-Co1-N42 75.45(9)<br>N30-Co1-N2 102.72(10)<br>N10-Co1-N2 75.27(10)<br>N22-Co1-N2 96.89(10)
**3**	Cd1-N2** 2.3501(14)<br>Cd1-N2** 2.3501(14)<br>Cd1-N10* 2.4177(14)<br>Cd1-N10* 2.4176(14)<br>Cd1-Cl21 2.5976(5)<br>Cd1-Cl21 2.5976(5)	N2-Cd1-N10 109.79(5)<br>N2-Cd1-N10 70.20(5)<br>N2-Cd1-N10 70.21(5)<br>N2-Cd1-N10 109.80(5)<br>N2-Cd1-Cl21 91.53(4)<br>N2-Cd1-Cl21 88.47(4)<br>N10-Cd1-Cl21 88.33(4)<br>N10-Cd1-Cl21 91.67(4)

		Cl21-Cd1-Cl21 180.0
**4**	Cu1-N30** 1.985(2)<br>Cu1-N50** 2.016(2)<br>Cu1-N10** 2.033(2)<br>Cu1-N22* 2.043(2)<br>Cu1-N42* 2.376(2)<br>Cu1-N2* 2.434(2)	N30-Cu1-N10 94.35(8)<br>N50-Cu1-N10 87.65(8)<br>N30-Cu1-N22 80.12(8)<br>N30-Cu1-N42 96.65(8)<br>N10-Cu1-N42 88.83(8)<br>N30-Cu1-N2 93.56(9)<br>N50-Cu1-N2 94.88(8)<br>N22-Cu1-N2 97.67(8)

***N**-donor atom from triazole ring from L; ****N**-donor atom from pyrazole ring from **L**

Table S2. Summary of crystallographic data and refinement for **1-4**.

Compounds	(**1**)	(**2**)	(**3**)	(**4**)
Empirical formula	$C_{21}H_{30}Cl_2N_{15}NiO_9$	$C_{21}H_{31}Cl_2CoN_{15}O_{10}$	C_{14}H18$CdCl_2N_{10}$	$C_{21}H_{27}CuN_{17}O_6$
Formula weight	766.16	783.44	509.68	677.13
Crystal size (mm)	0.19 x 0.12 x 0.04	0.40 x 0.35 x 0.28	0.80 x 0.50 x 0.40	0.30 x 0.20 x 0.15
Crystal system	hexagonal	triclinic	triclinic	monoclinic
Space group	$P\ 6_3/m$ (#176)	*P*-1 (#2)	*P*-1 (#2)	$P\ 2_1/c$ (#14)
a (Å)	14.3552(4)	10.4484(16)	7.08300(10)	18.9613(9)
b (Å)	14.3552(4)	12.6439(19)	8.0964(2)	9.9100(5)
c (Å)	22.0836(7)	14.6686(17)	9.0990(6)	16.1636(8)
α (°)	90	107.929(12)	78.067(6)	90
β (°)	90	103.062(12)	86.157(6)	97.988(5)
γ (°)	120	105.385(13)	66.889(5)	90
Volume (Å^3)	3941.1(2)	1674.7(4)	469.49(4)	3007.8(3)
Z	4	2	1	4
ρcalc.(g/cm^3)	1.459	1.554	1.803	1.495
F(000)	1776	806	254	1396
μMoK_α (cm^{-1})	0.769	0.746	1.471	0.793
T (K)	293(2)	293(2)	293	297(2)
θ min/max	2.984/26.183	3.039/26.020	3.127/ 27.469	3.107/26.072
Refl.collected/unique	28234/2697	27221/6565	7335/2152	22844/5912
Data/restraints/parameters	2697/82/221	6565/208/546	2152/0/126	5912/0/412
Goodness of fit on F^2	1.091	1.077	1.150	1.045

Final *R* indices [I>2σ(I)]	R_1=0.0744, wR_2=0.1933	R_1=0.0595, wR_2=0.1575	R_1 = 0.0233, wR_2 = 0.0586	R_1=0.0397, wR_2=0.1029
R indices (all data)	R_1= 0.0808, wR_2=0.1975	R_1= 0. 0676, wR_2=0.1636	R_1 = 0.0234, wR_2 = 0.0586	R_1= 0. 0525, wR_2=0.1091

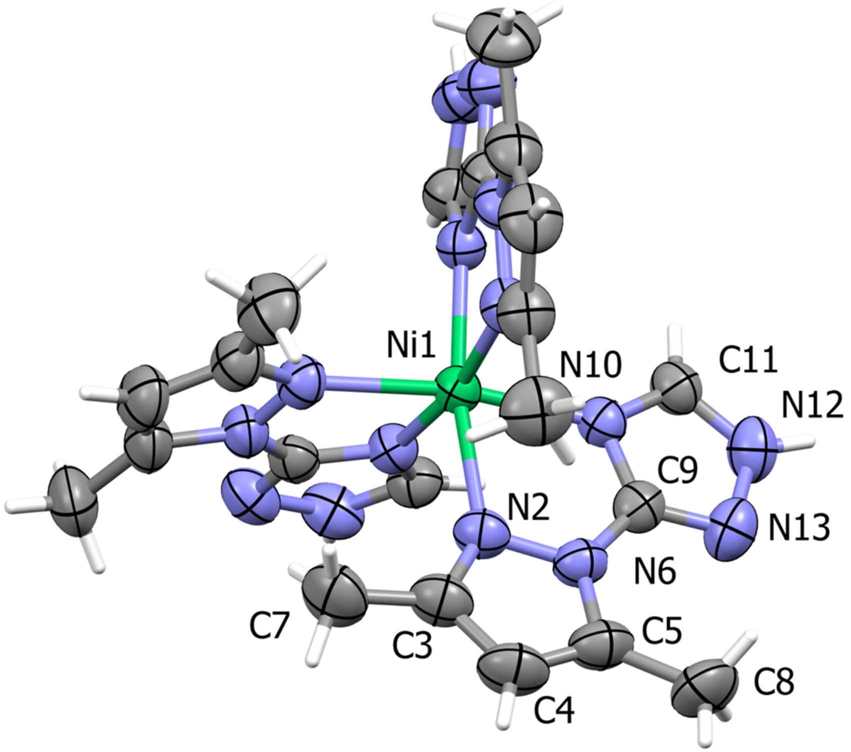

Figure S1. Molecular crystal structures for $(H_3O)\{[NiL_3](ClO_4)_3\}$ (**1**), displacement ellipsoids drawn at the 50% probability level and atom numbering scheme. The ClO_4^- anion and H_3O cation were omitted for clarity.

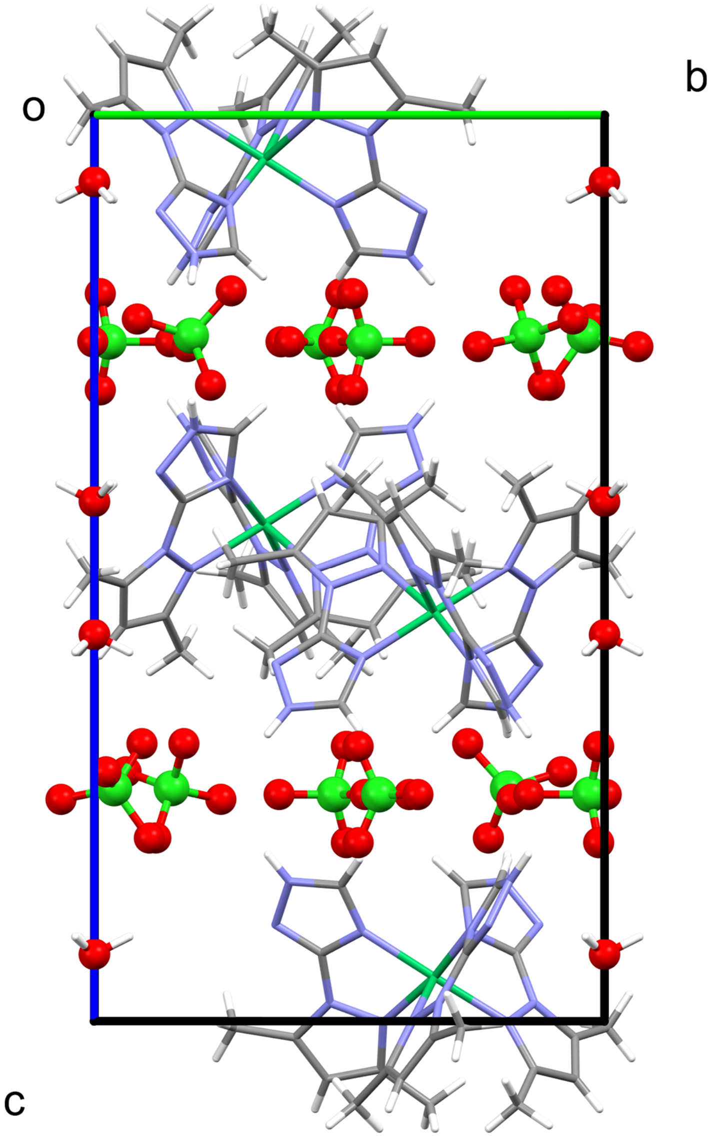

Figure S2. Stick representation of the unit cell packing of **1**, shown along the a-axis. Anions are shown in ball and stick style. The minor parts of the disordered molecules were omitted for clarity.

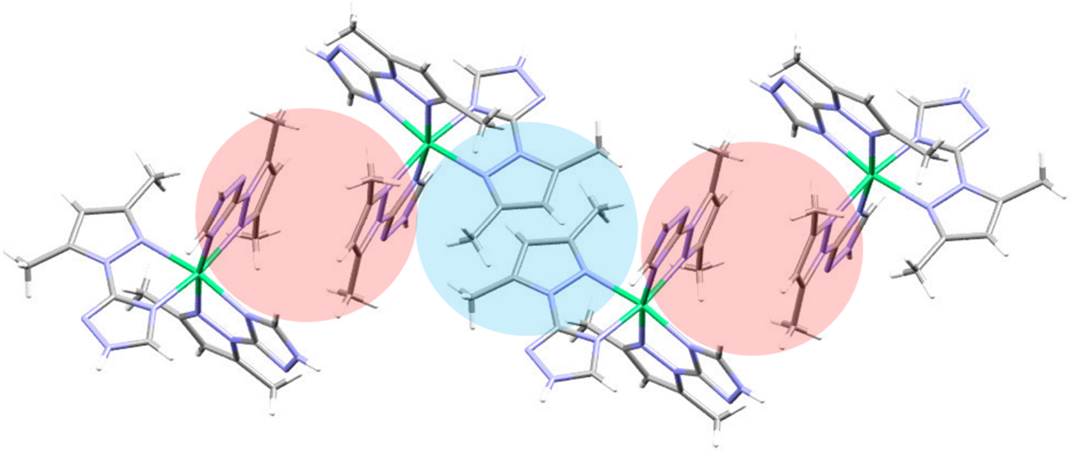

Figure S3. Packing details for **1** showing the π-π stacking interaction between pyrazole rings from neighboring complex molecules.

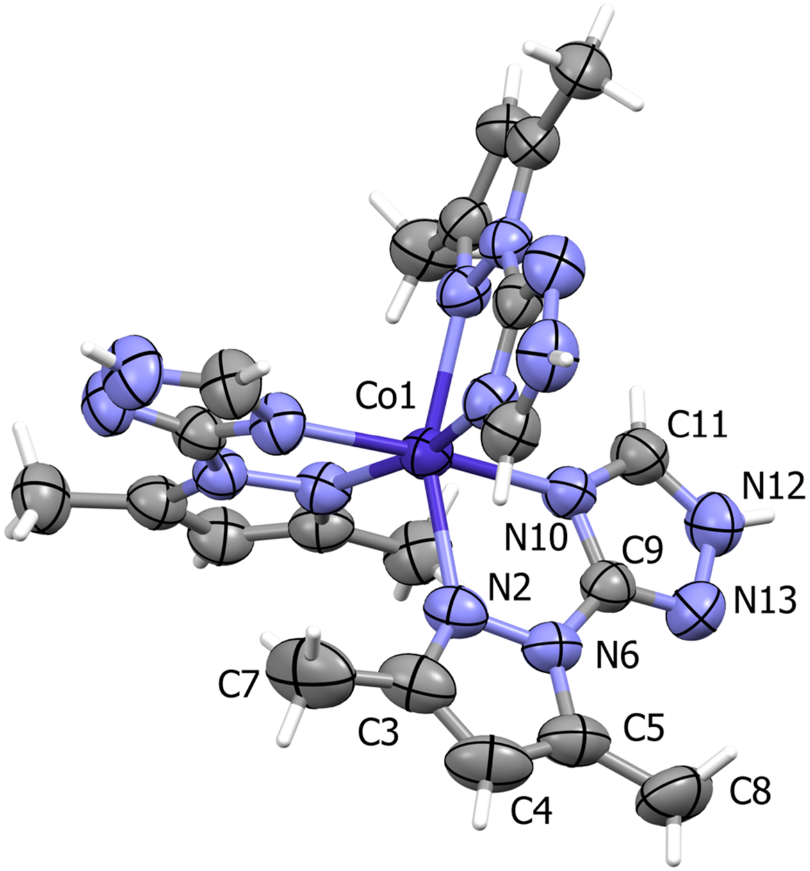

Figure S4. Molecular crystal structures for $[CoL_3](ClO_4)_2 \cdot 2H_2O$ (**2**), displacement ellipsoids drawn at the 50% probability level and atom numbering scheme. The ClO_4^- anions and H_2O molecule were omitted for clarity. Atom numbering for the other ligands is incremented by 20.

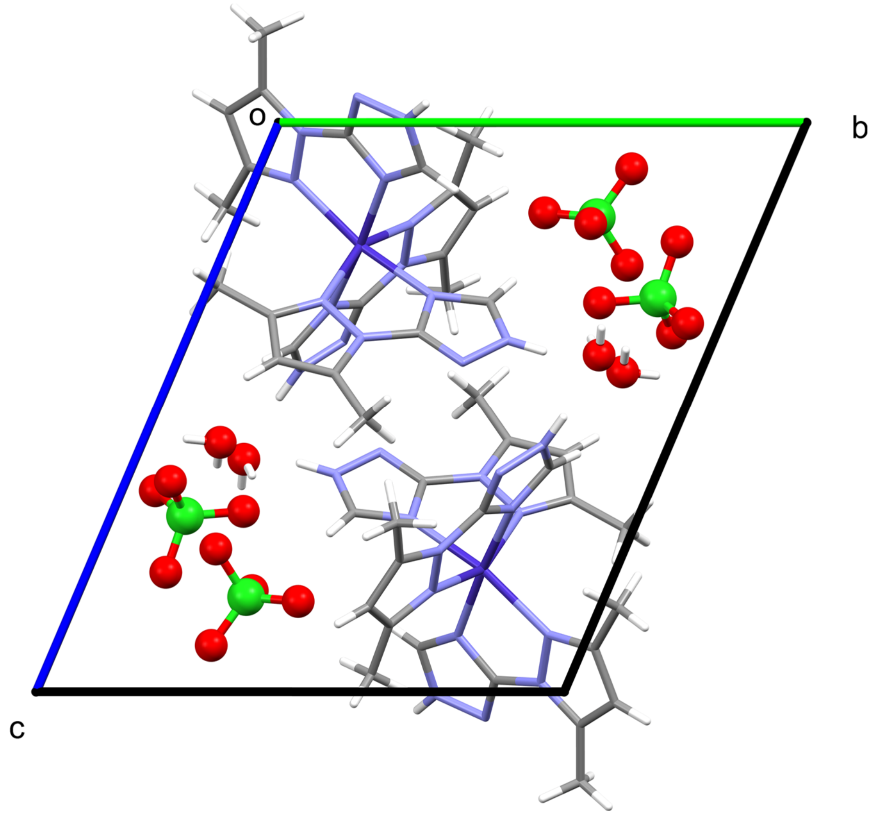

Figure S5. Stick representation of the unit cell packing of **2**, shown along the *a*-axis. Anions are shown in ball and stick style. The minor parts of the disordered molecules were omitted for clarity.

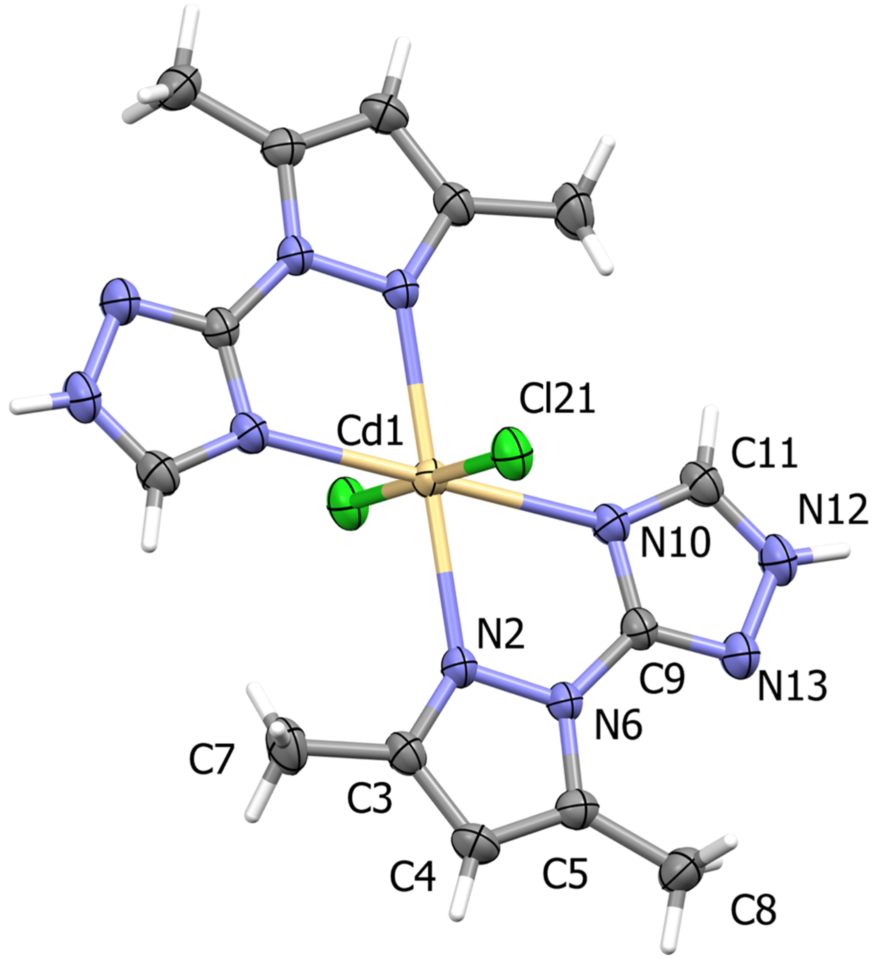

Figure S6. Molecular crystal structures for [CdL_2Cl_2] (**3**), displacement ellipsoids drawn at the 50% probability level and atom numbering scheme.

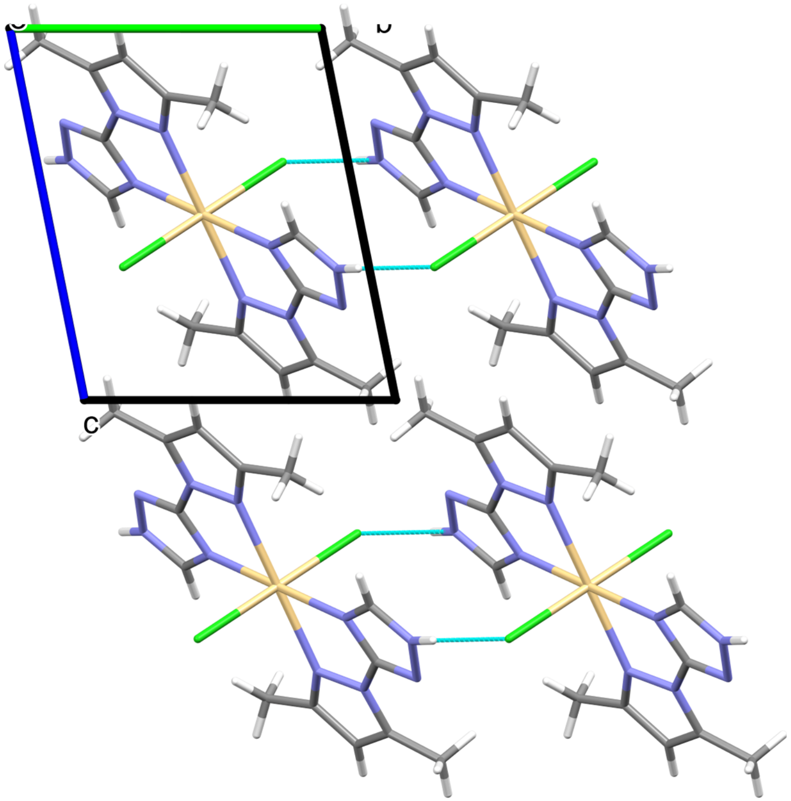

Figure S7. Stick representation of the packing of **3**, shown along the *a*-axis.

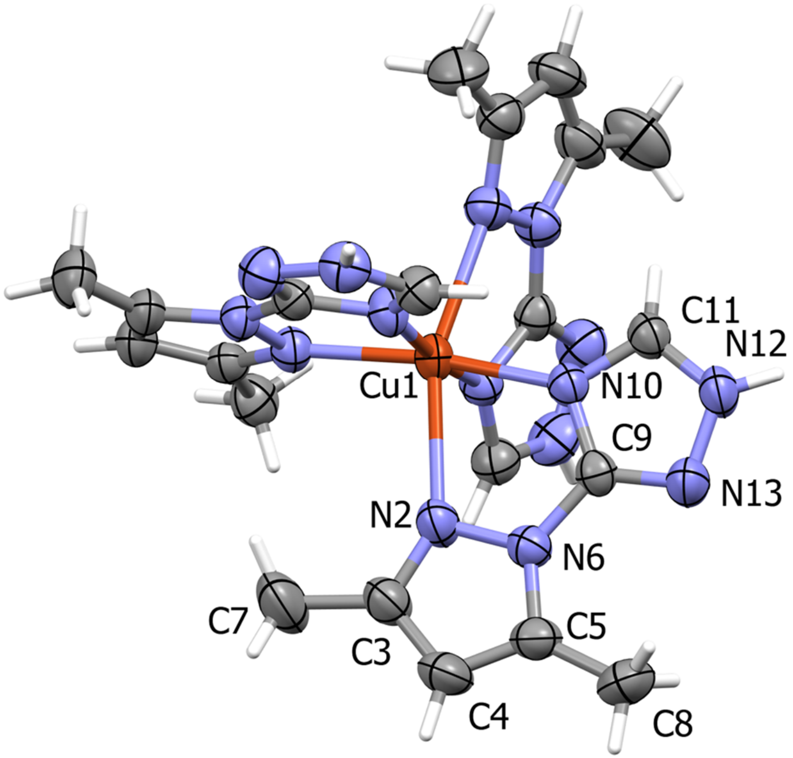

Figure S8. Molecular crystal structures for $[CuL_3](NO_3)_2$ (**4**), displacement ellipsoids drawn at the 50% probability level and atom numbering scheme. Anions were omitted for clarity. Atom numbering for the other ligands is incremented by 20.

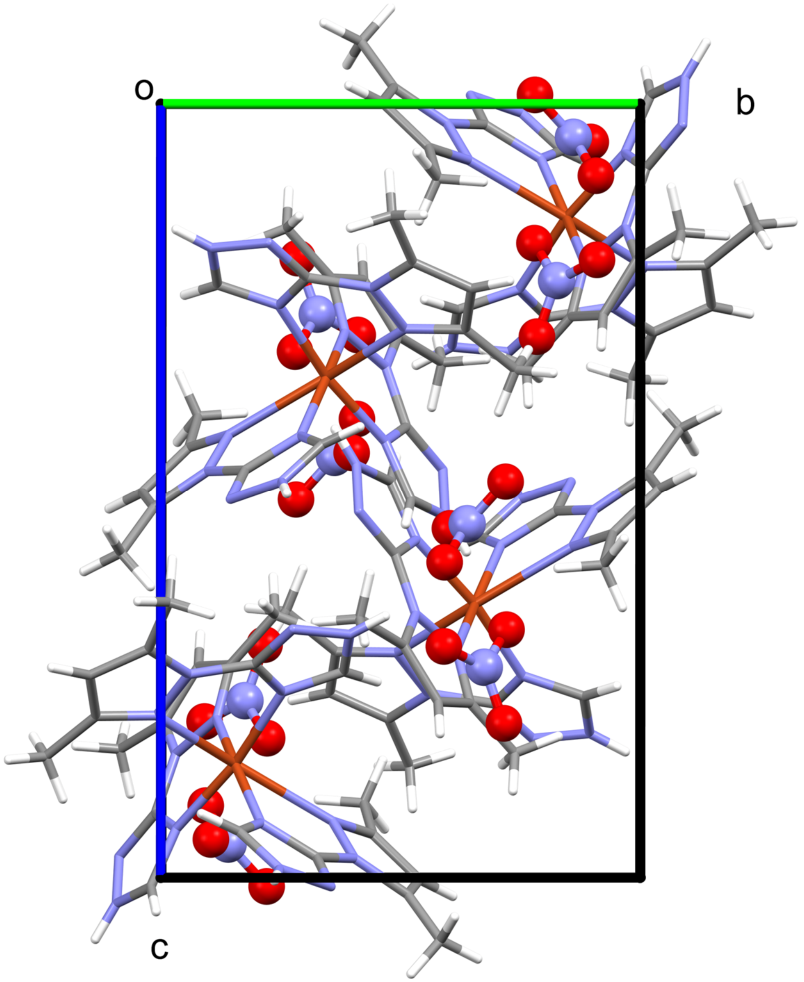

Figure S9. Stick representation of the unit cell packing of **4**, shown along the *a*-axis. Anions are shown in ball and stick style.

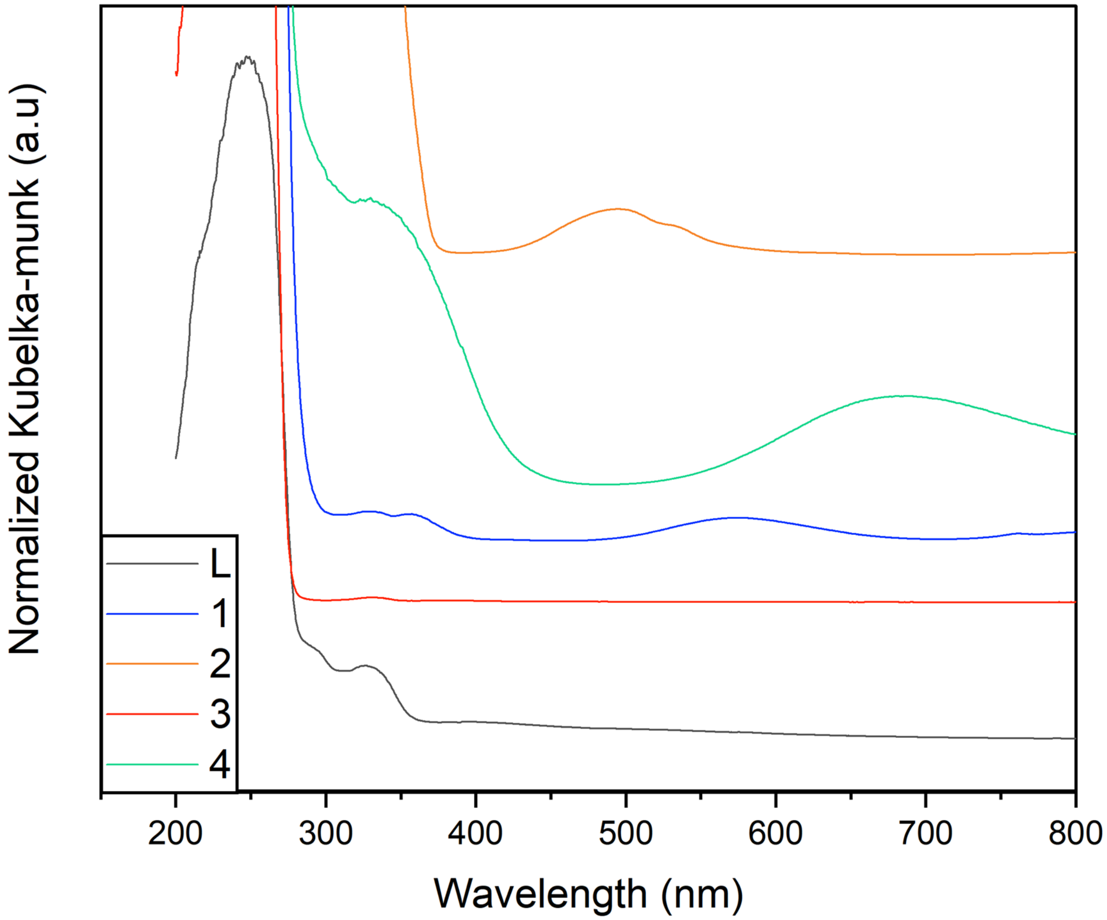

Figure S10. Diffuse reflectance spectroscopy comparison plot of **L** with **1- 4**

^{1}H NMR spectrum of L:

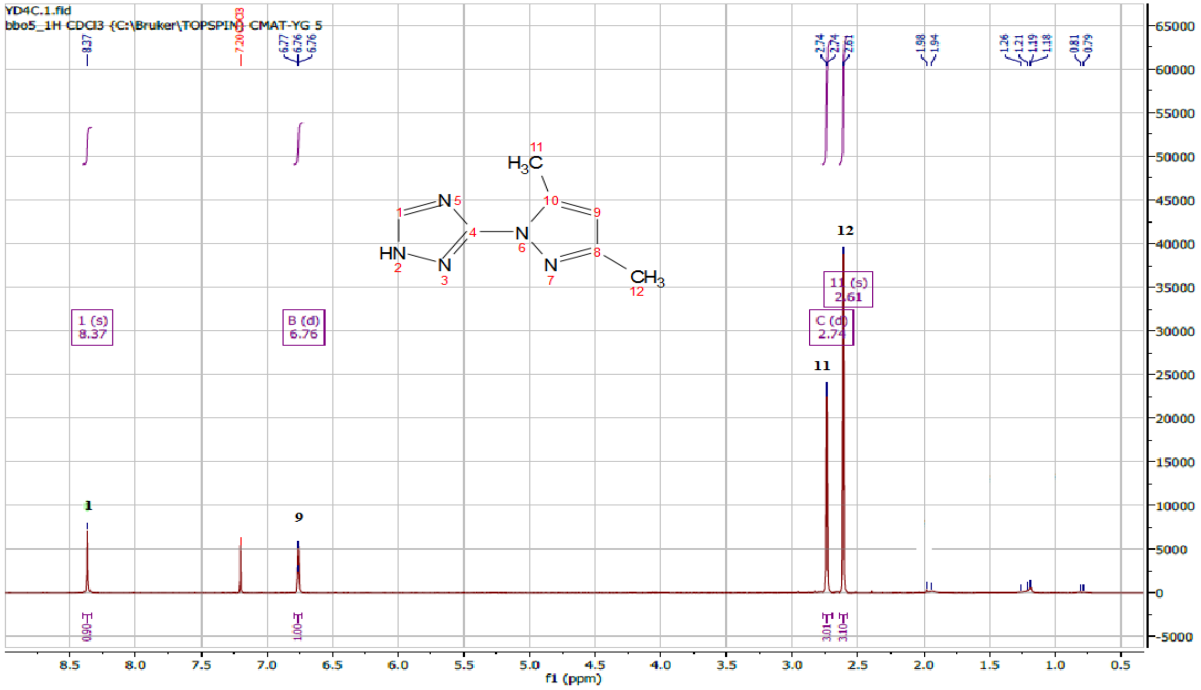

^{13}C NMR spectrum of L:

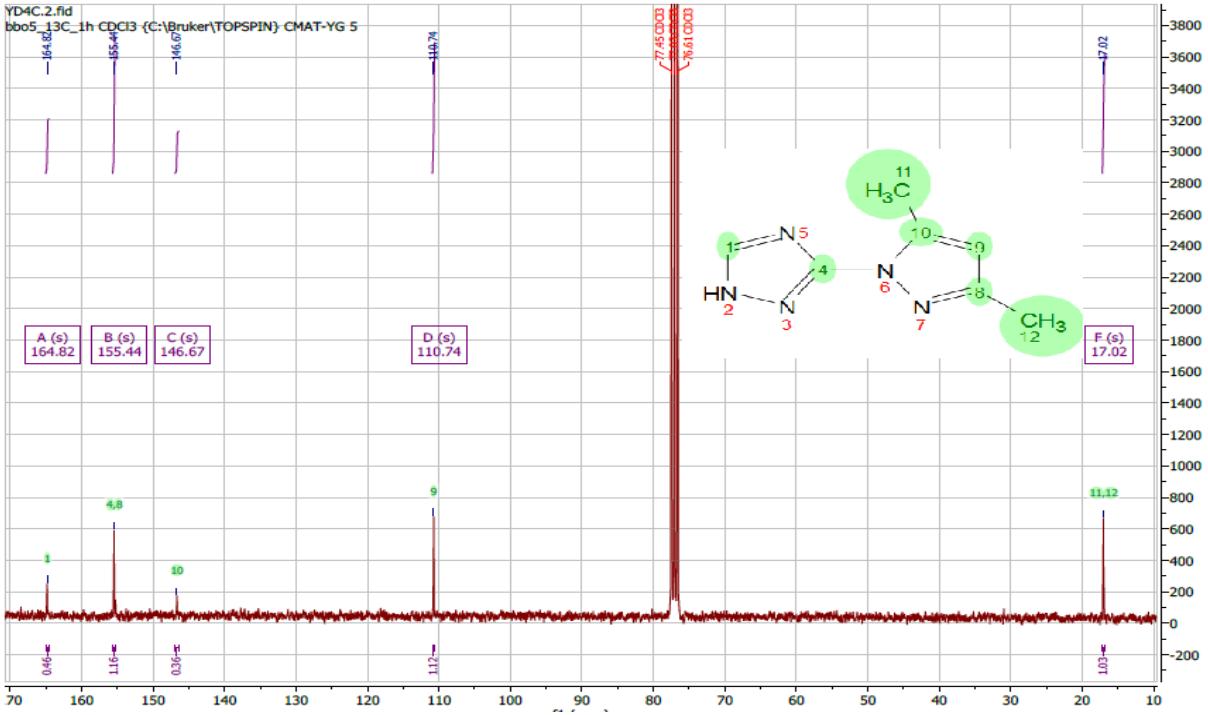

^{1}H NMR spectrum of 3:

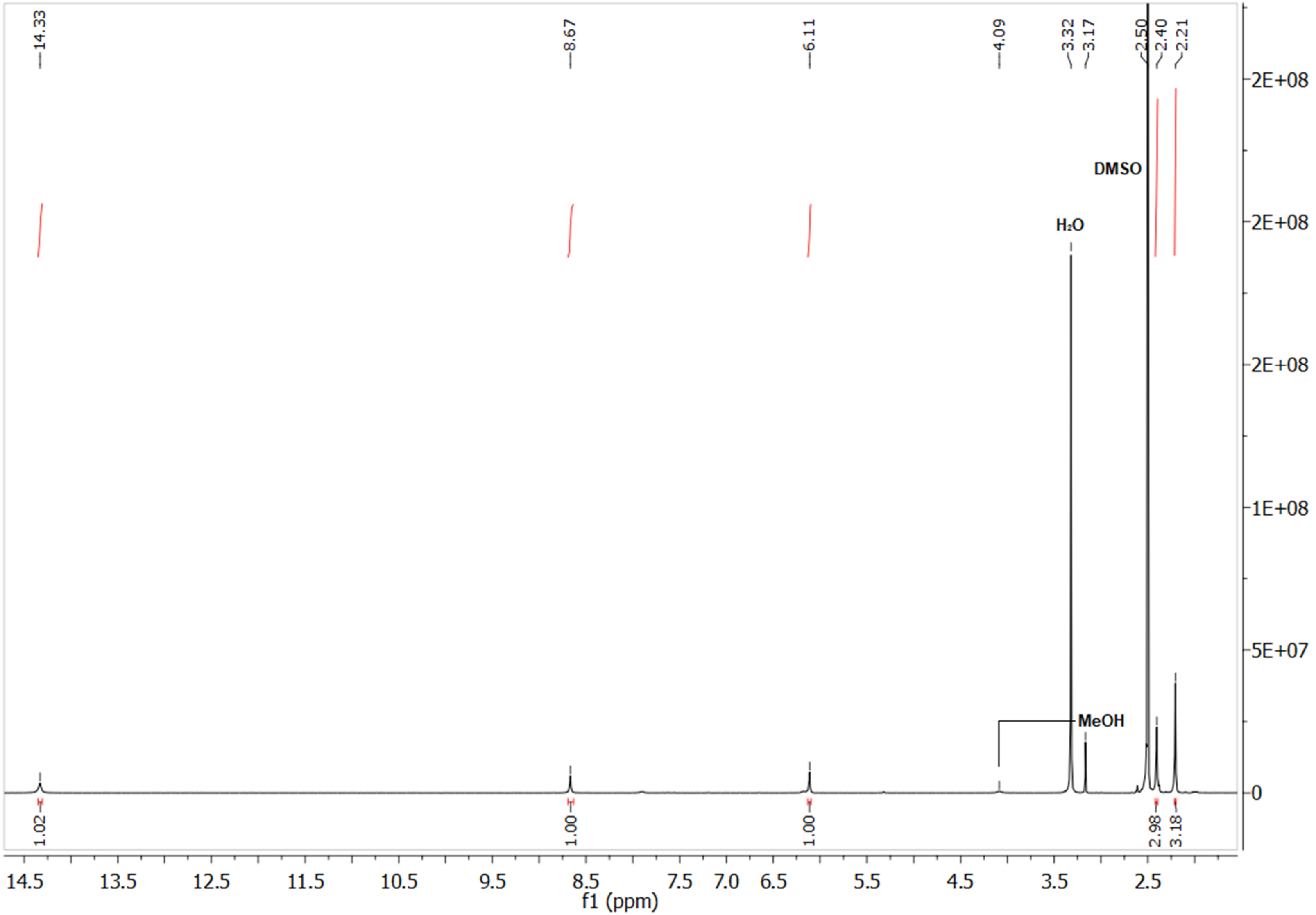
14.33
8.67
6.11
4.09
3.32
3.17
2.50
2.40
2.21
DMSO
H₂O
MeOH
1.02
1.00
1.00
2.98
3.18
2E+08
2E+08
2E+08
1E+08
5E+07
0
14.5 13.5 12.5 11.5 10.5 9.5 8.5 7.5 7.0 6.5 5.5 4.5 3.5 2.5
f1 (ppm)

^{13}C NMR spectrum of 3:

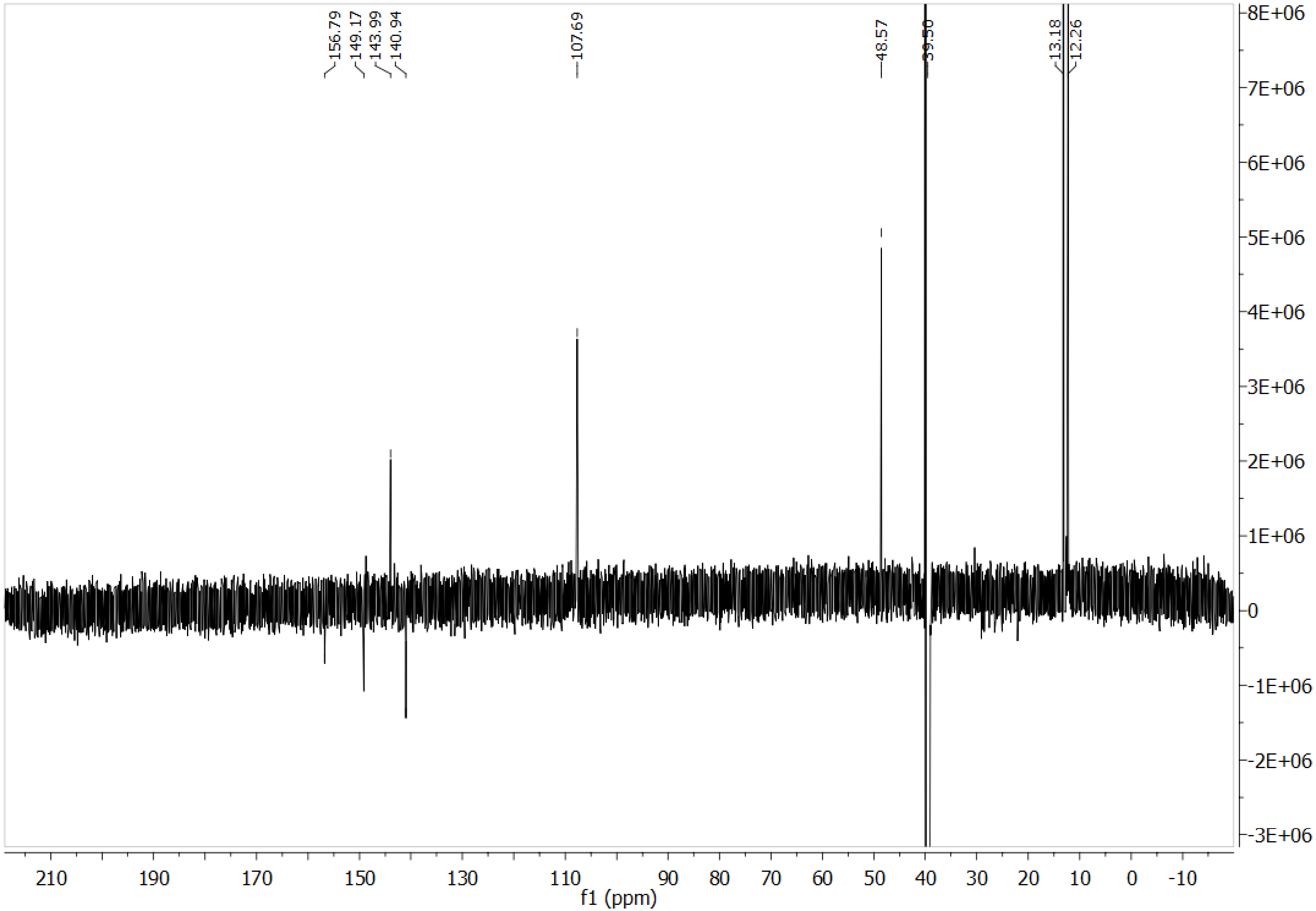

HRMS of ligand L

ESI (+), MeCN/MeOH + 1% H_2O

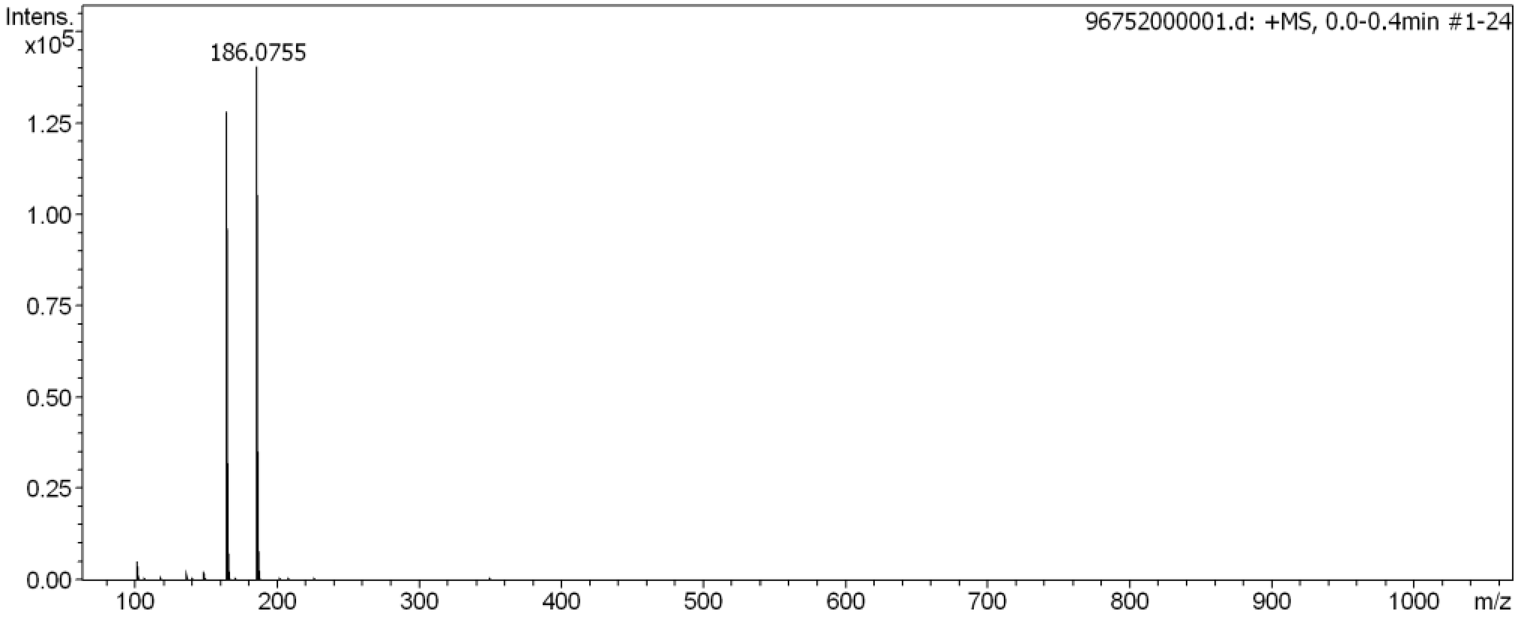

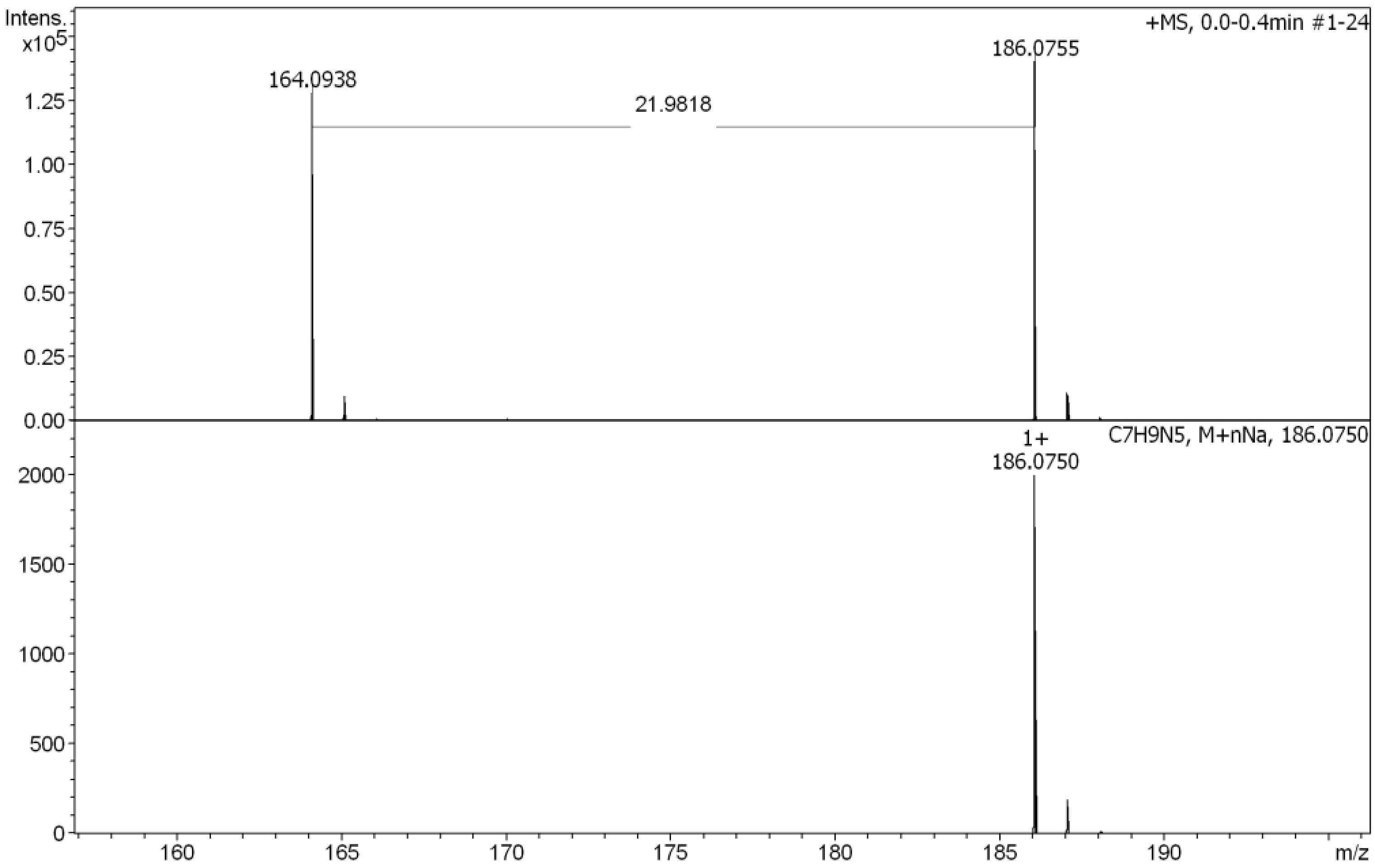

Intens.
x10^5
+MS, 0.0-0.4min #1-24
164.0938
186.0755
21.9818
1.25
1.00
0.75
0.50
0.25
0.00
C7H9N5, M+nNa, 186.0750
1+
186.0750
2000
1500
1000
500
0
160
165
170
175
180
185
190
m/z

HRMS of 1

ESI (+), MeCN/MeOH + 1% H_2O

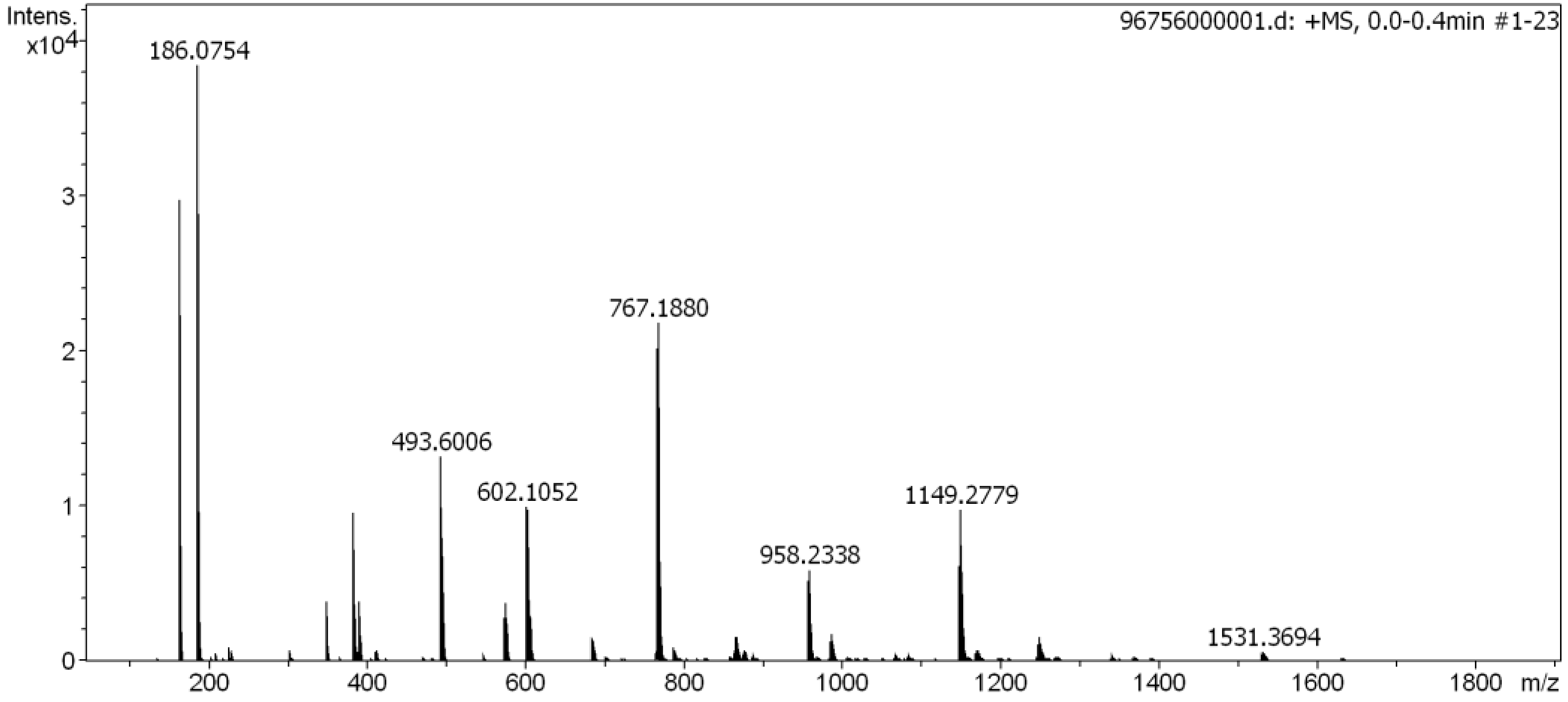

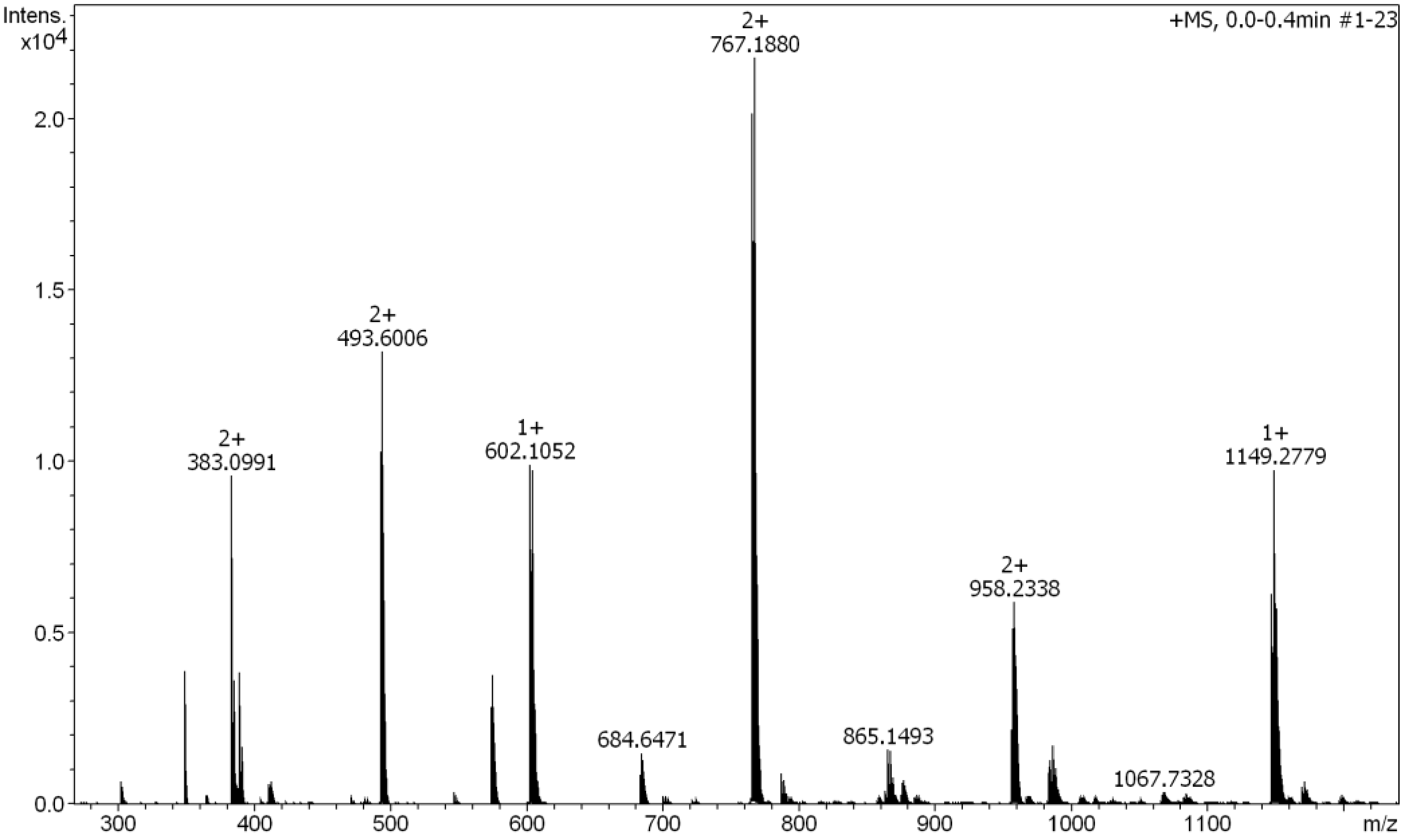
Intens.
x10^4
+MS, 0.0-0.4min #1-23
2+
767.1880
2+
493.6006
2+
383.0991
1+
602.1052
1+
1149.2779
2+
958.2338
684.6471
865.1493
1067.7328
2.0
1.5
1.0
0.5
0.0
300
400
500
600
700
800
900
1000
1100
m/z

HRMS of 1

ESI (–), MeCN/MeOH + 1% H_2O

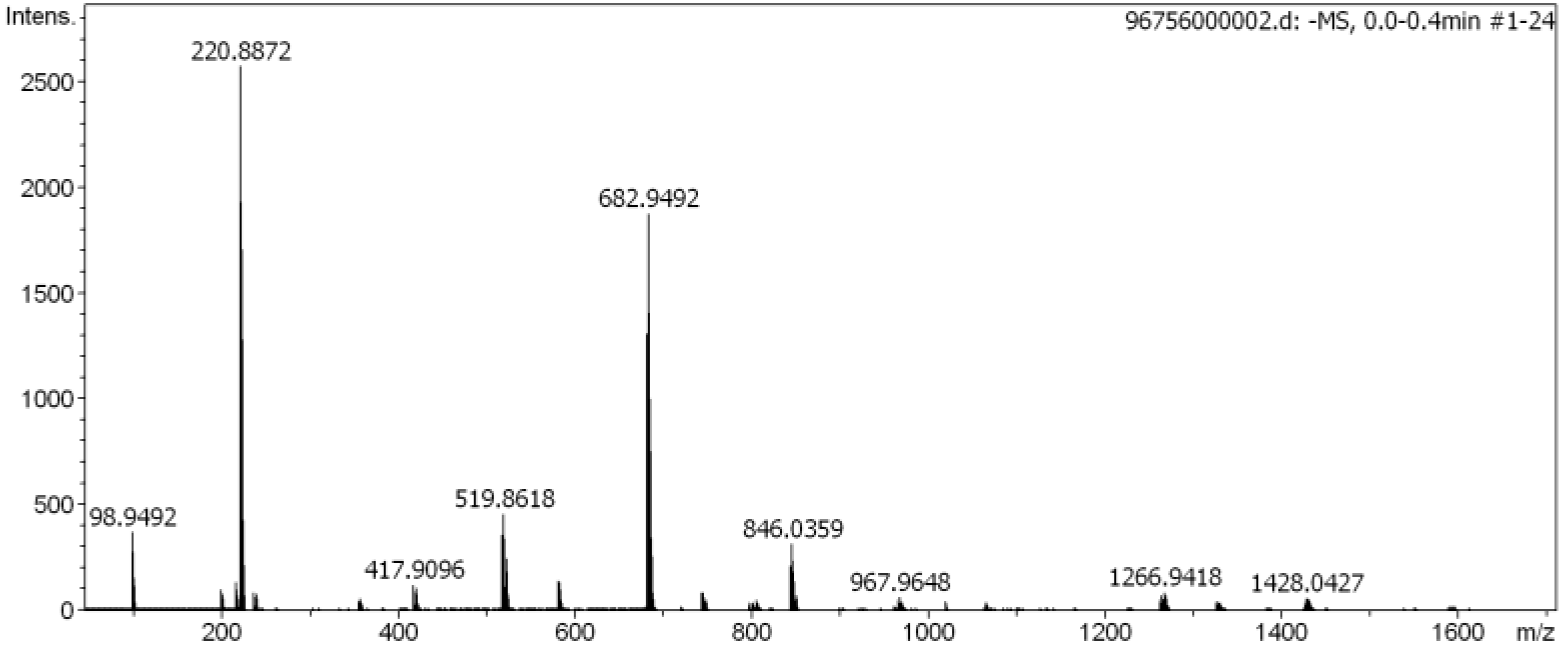

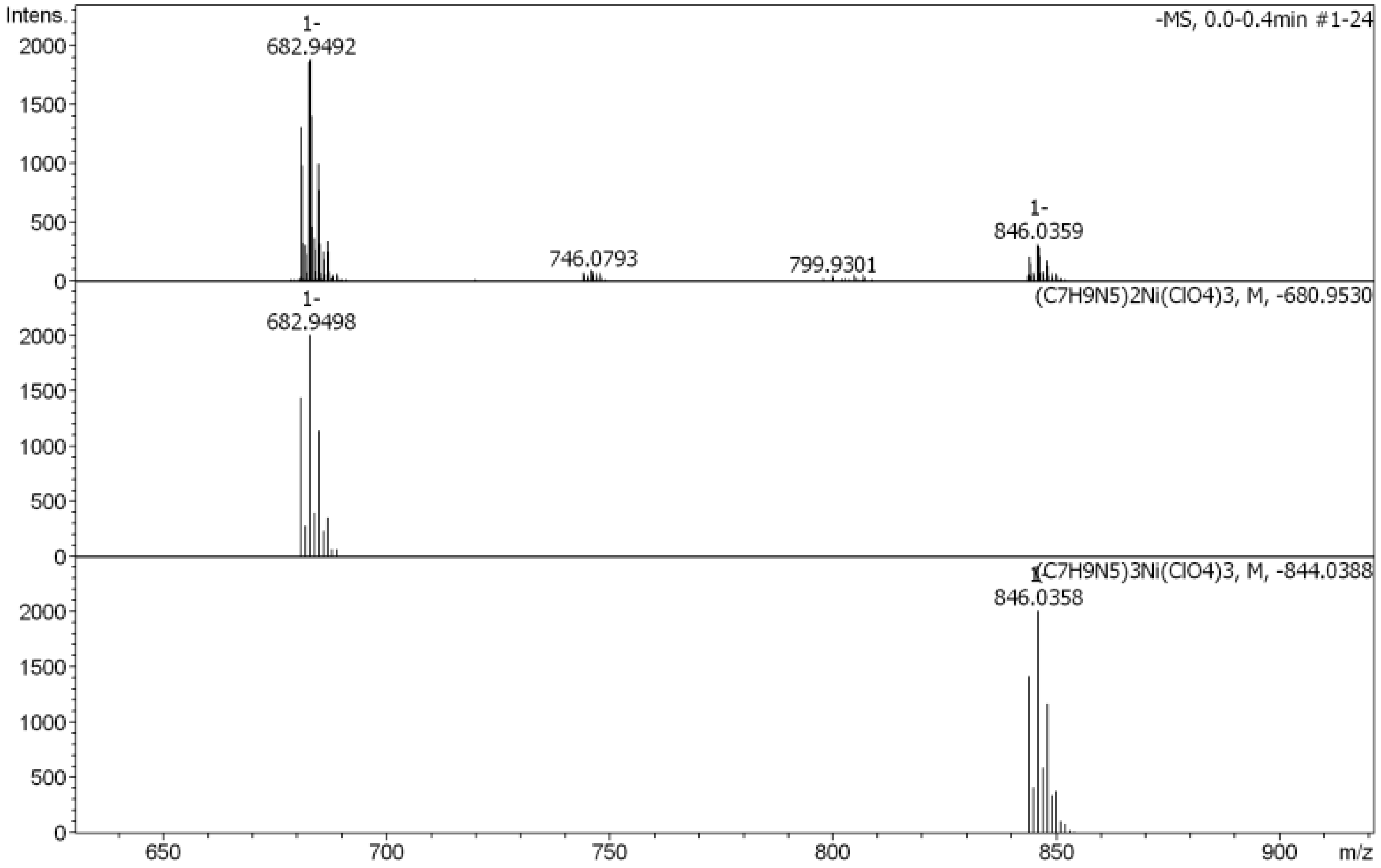
Intens.
-MS, 0.0-0.4min #1-24
1-
682.9492
746.0793
799.9301
1-
846.0359
(C7H9N5)2Ni(ClO4)3, M, -680.9530
1-
682.9498
(C7H9N5)3Ni(ClO4)3, M, -844.0388
1-
846.0358
m/z

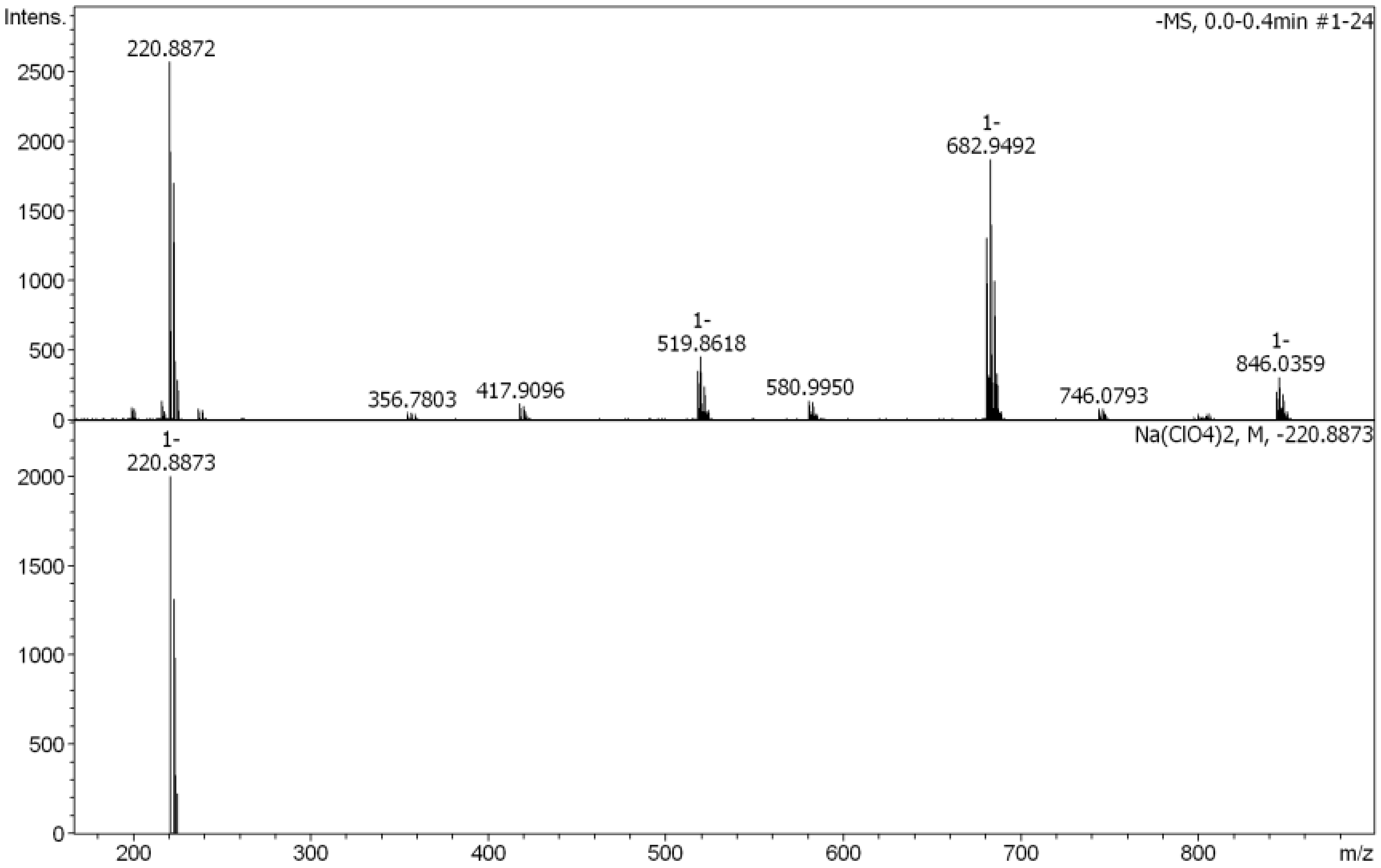
Intens.
-MS, 0.0-0.4min #1-24
220.8872
1-
682.9492
1-
519.8618
1-
846.0359
356.7803
417.9096
580.9950
746.0793
Na(ClO4)2, M, -220.8873
1-
220.8873
2500
2000
1500
1000
500
0
200
300
400
500
600
700
800
m/z

HRMS of 2

ESI (+), MeCN/MeOH + 1% H_2O

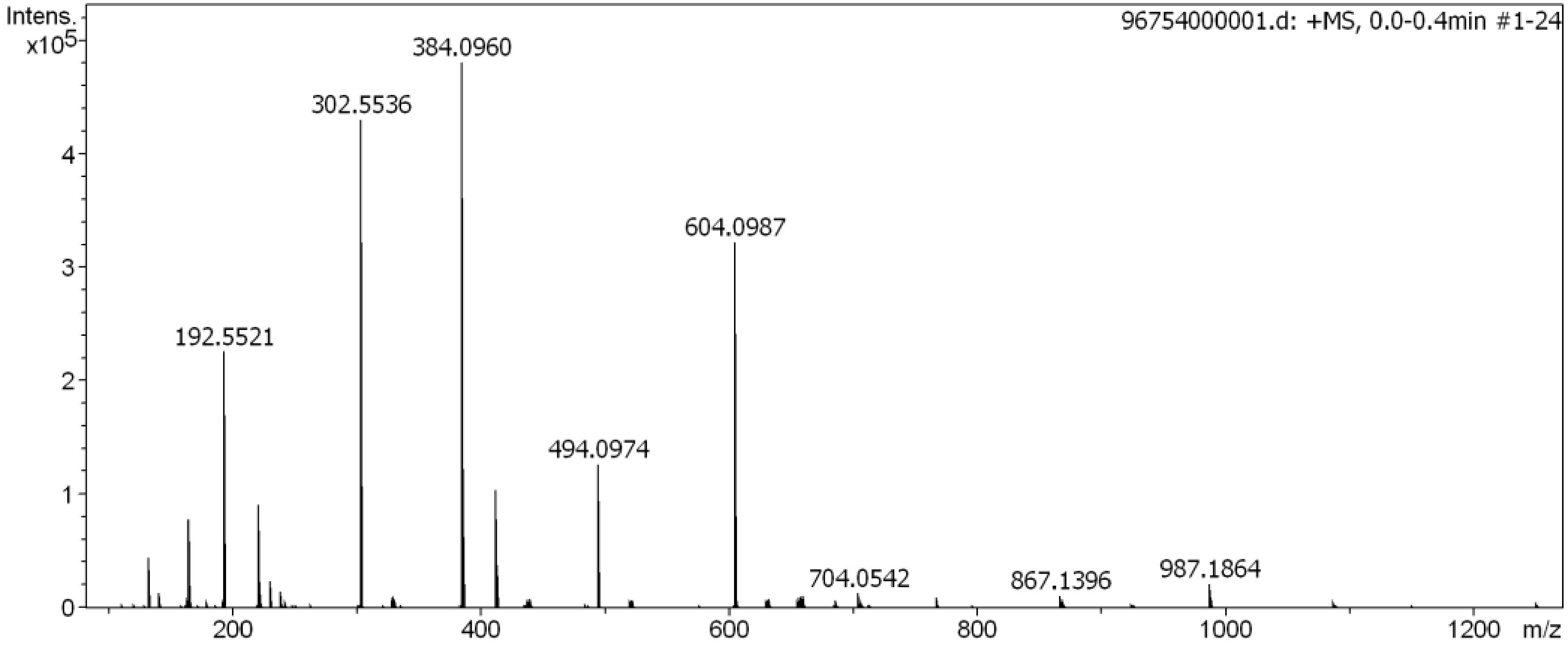

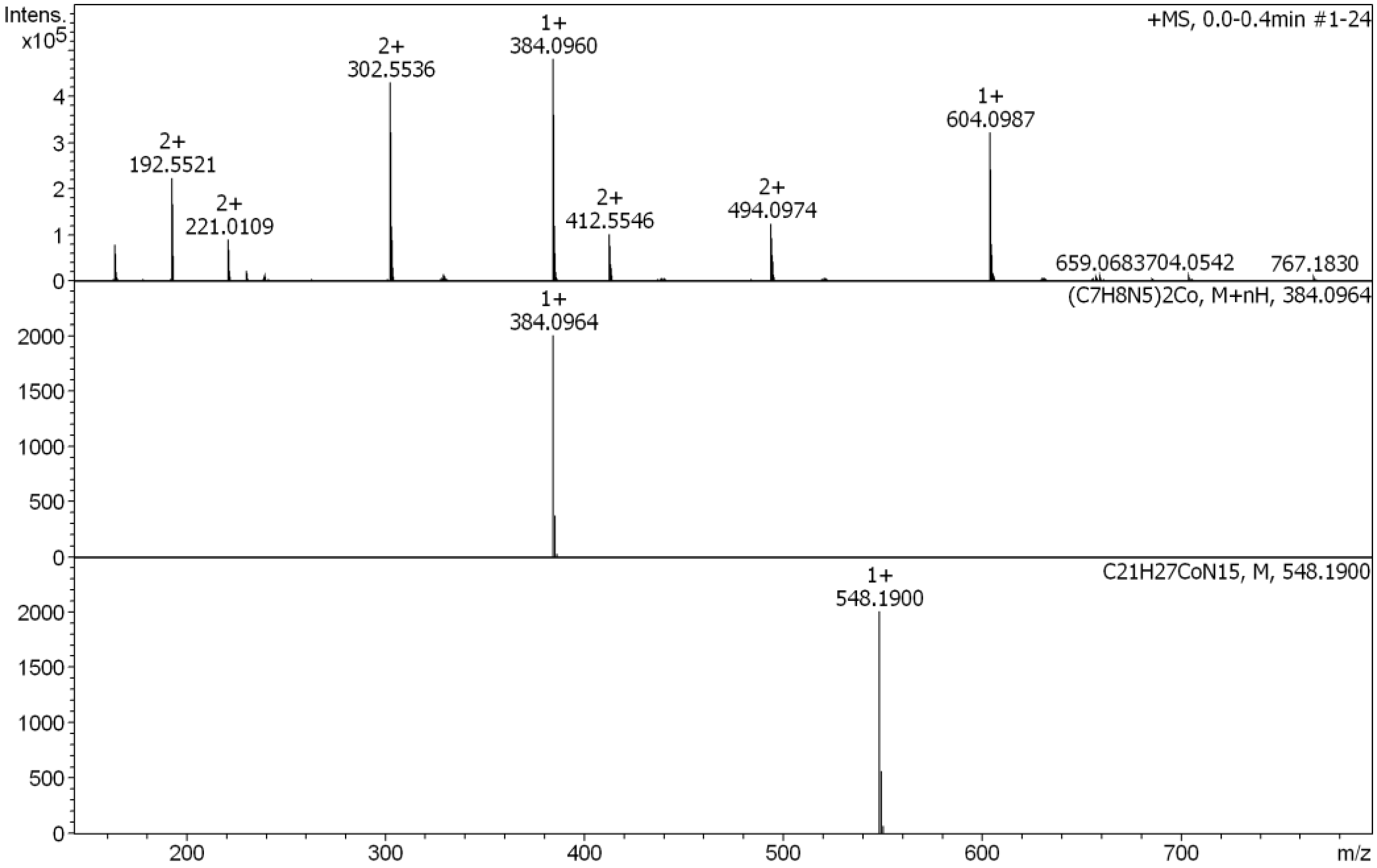
Intens.
x10^5
+MS, 0.0-0.4min #1-24
2+
192.5521
2+
221.0109
2+
302.5536
1+
384.0960
2+
412.5546
2+
494.0974
1+
604.0987
659.0683
704.0542
767.1830
(C7H8N5)2Co, M+nH, 384.0964
1+
384.0964
C21H27CoN15, M, 548.1900
1+
548.1900
m/z

HRMS of 2

ESI (-), MeCN/MeOH + 1% H_2O

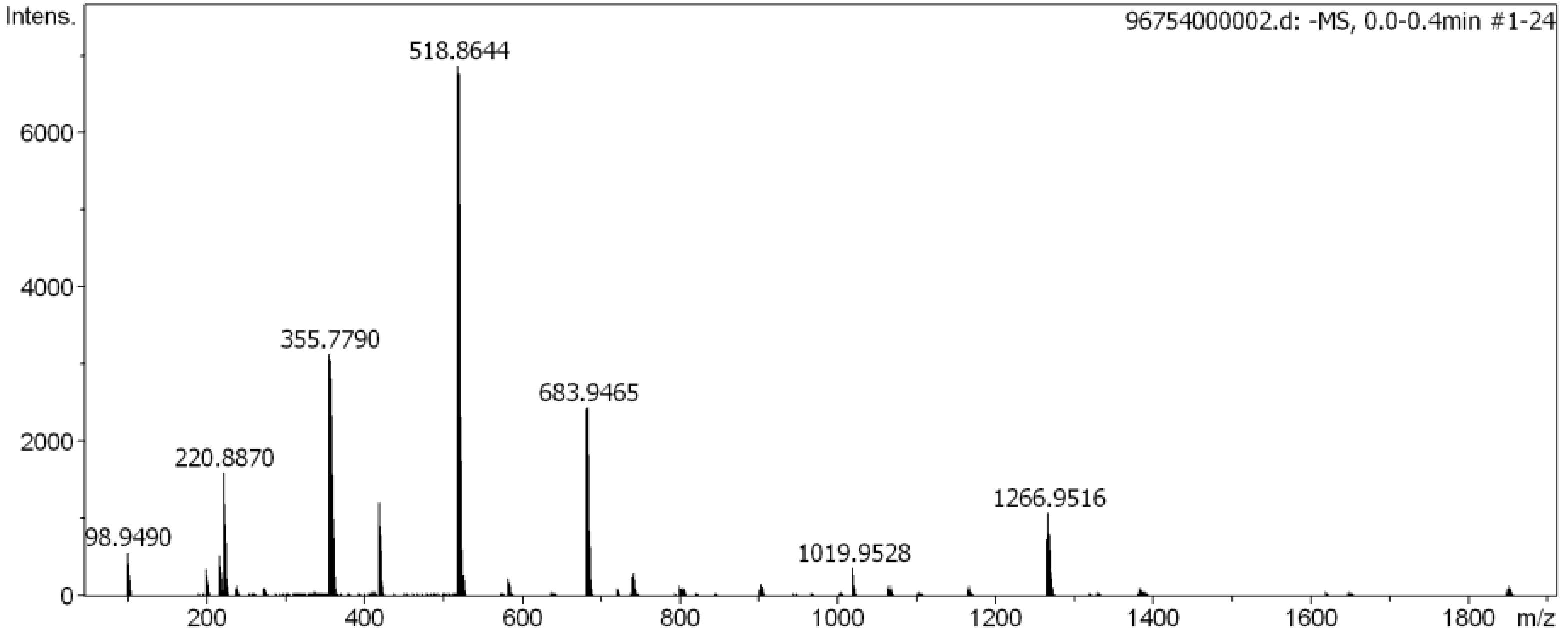

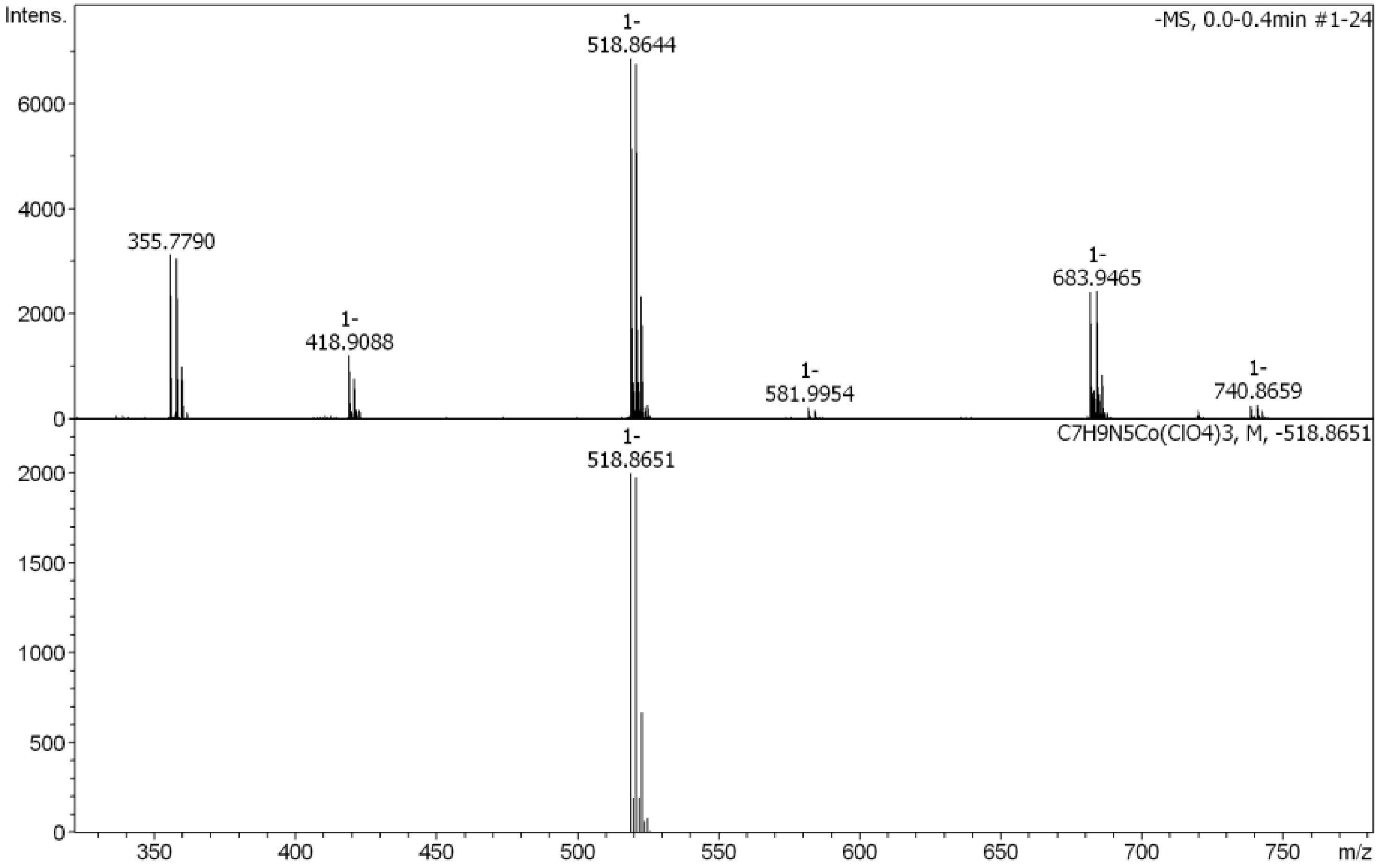

Intens.
-MS, 0.0-0.4min #1-24
1-
518.8644
355.7790
1-
418.9088
1-
581.9954
1-
683.9465
1-
740.8659
C7H9N5Co(ClO4)3, M, -518.8651
1-
518.8651
6000
4000
2000
0
2000
1500
1000
500
0
350
400
450
500
550
600
650
700
750
m/z

HRMS of 3

ESI (+), MeCN/MeOH + 1% H_2O

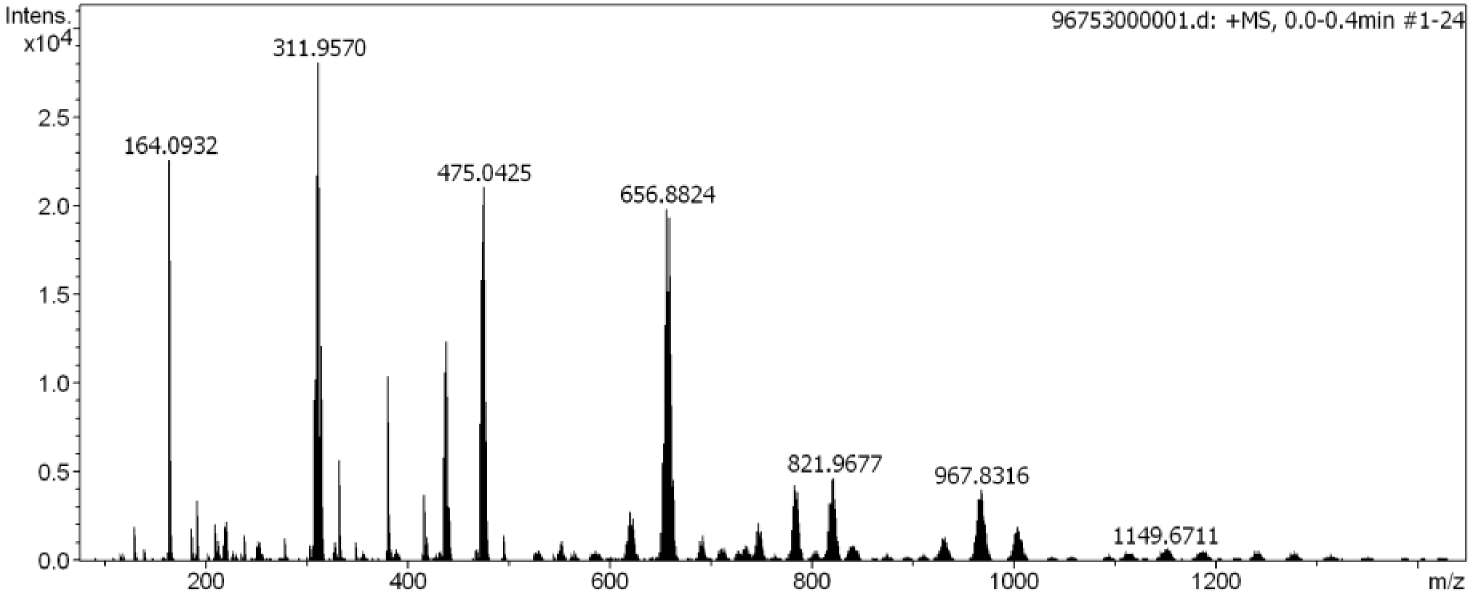

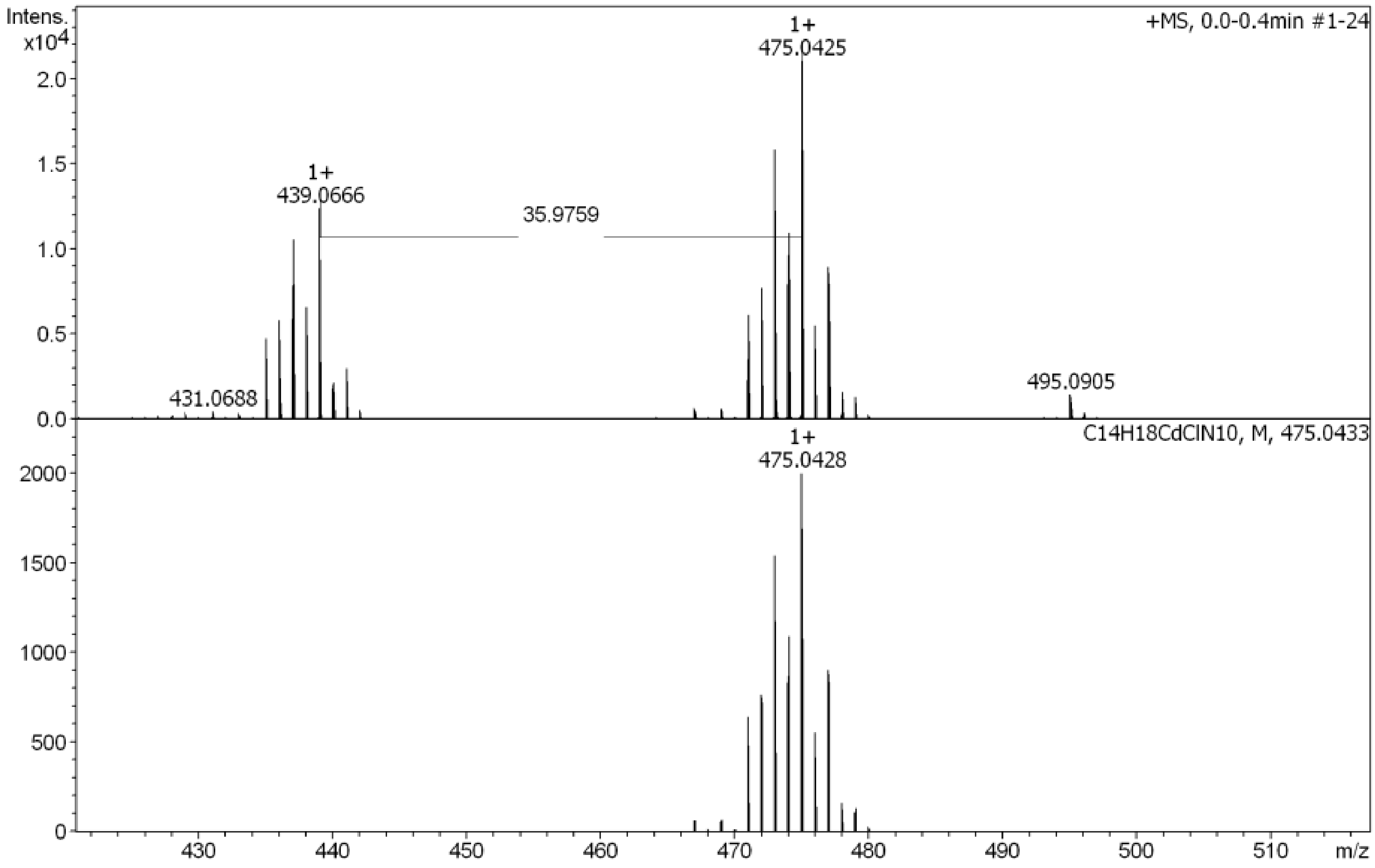
Intens.
x10^4
+MS, 0.0-0.4min #1-24
1+
475.0425
1+
439.0666
35.9759
431.0688
495.0905
C14H18CdClN10, M, 475.0433
1+
475.0428
m/z

HRMS of 3

ESI (-), MeCN/MeOH + 1% H_2O

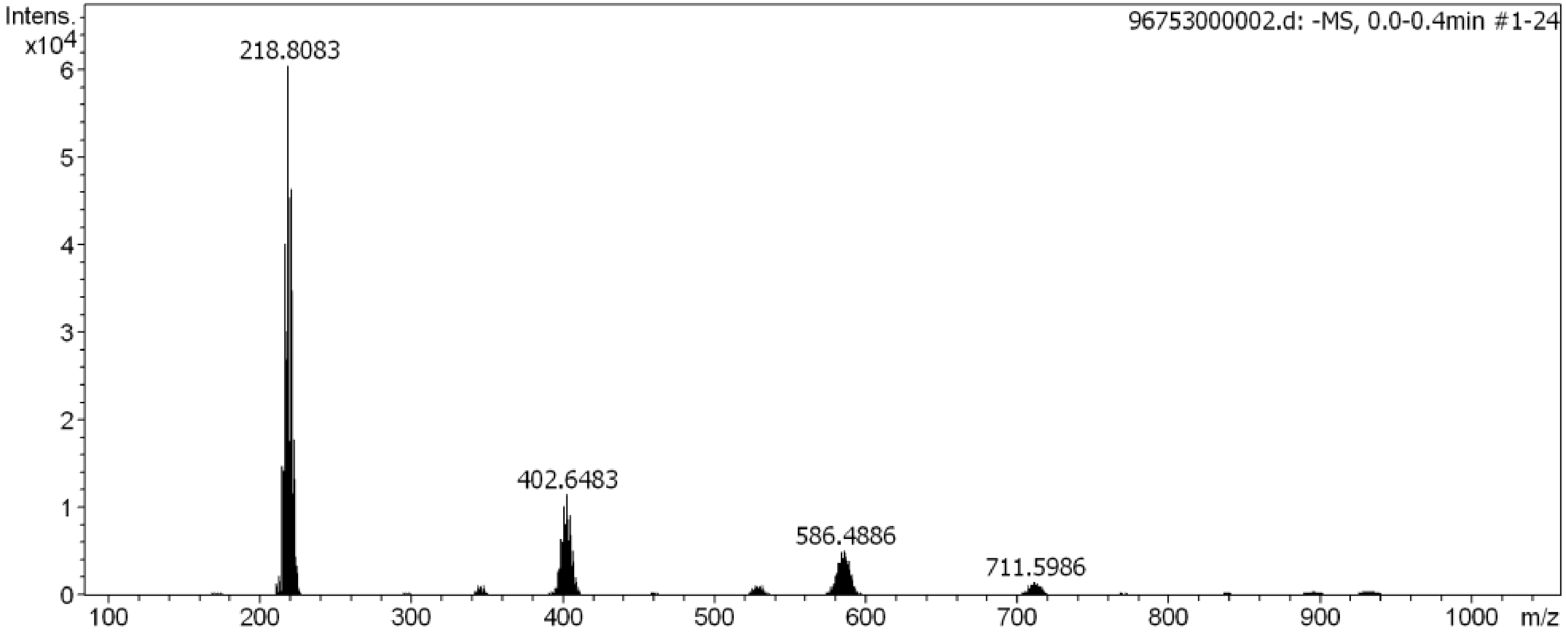

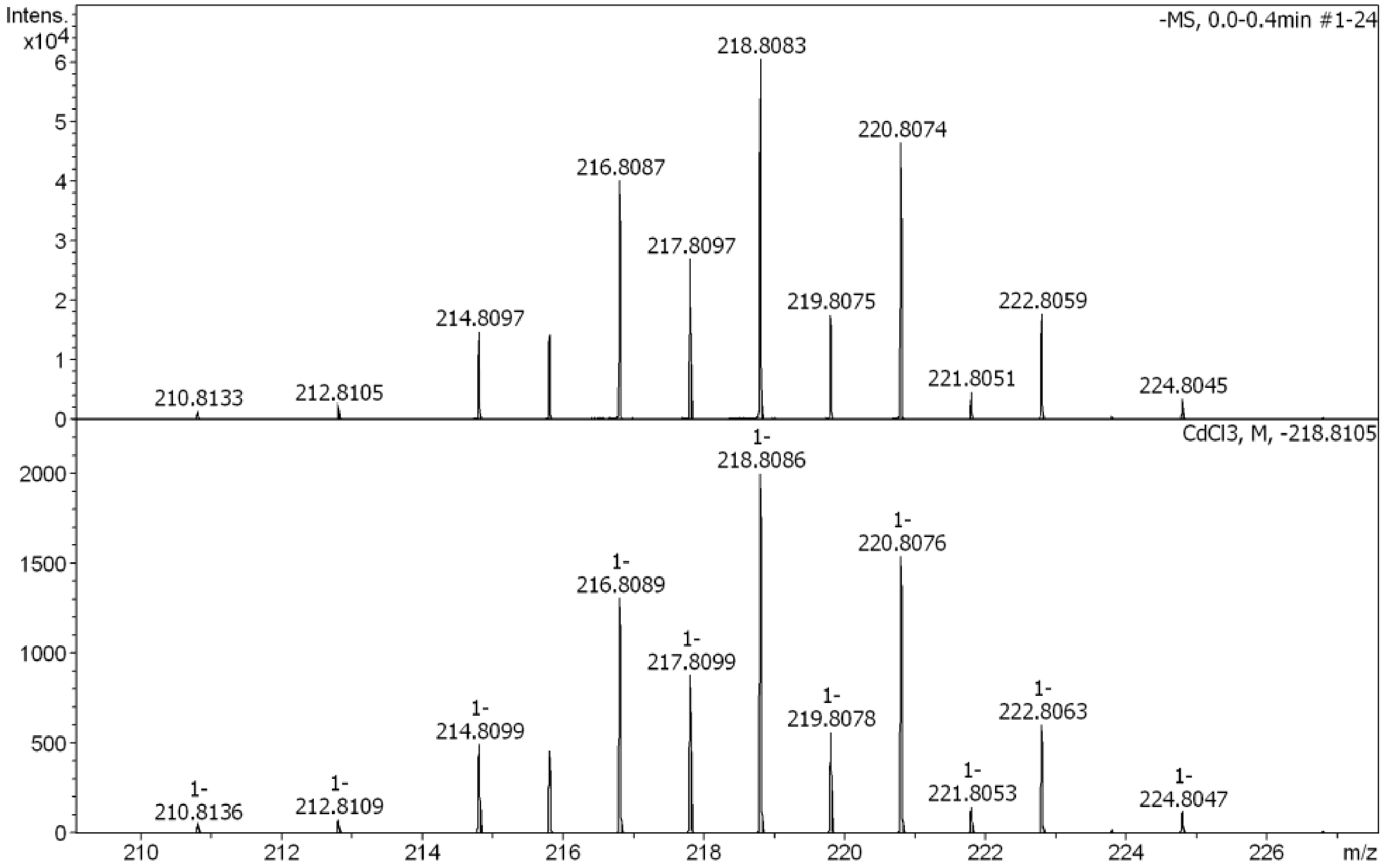
Intens.
x10^4
-MS, 0.0-0.4min #1-24
218.8083
220.8074
216.8087
217.8097
219.8075
222.8059
214.8097
221.8051
224.8045
210.8133
212.8105
CdCl3, M, -218.8105
1-
218.8086
1-
220.8076
1-
216.8089
1-
217.8099
1-
219.8078
1-
222.8063
1-
214.8099
1-
221.8053
1-
224.8047
1-
210.8136
1-
212.8109
m/z

HRMS of 4

ESI (+), MeCN/MeOH + 1% H_2O

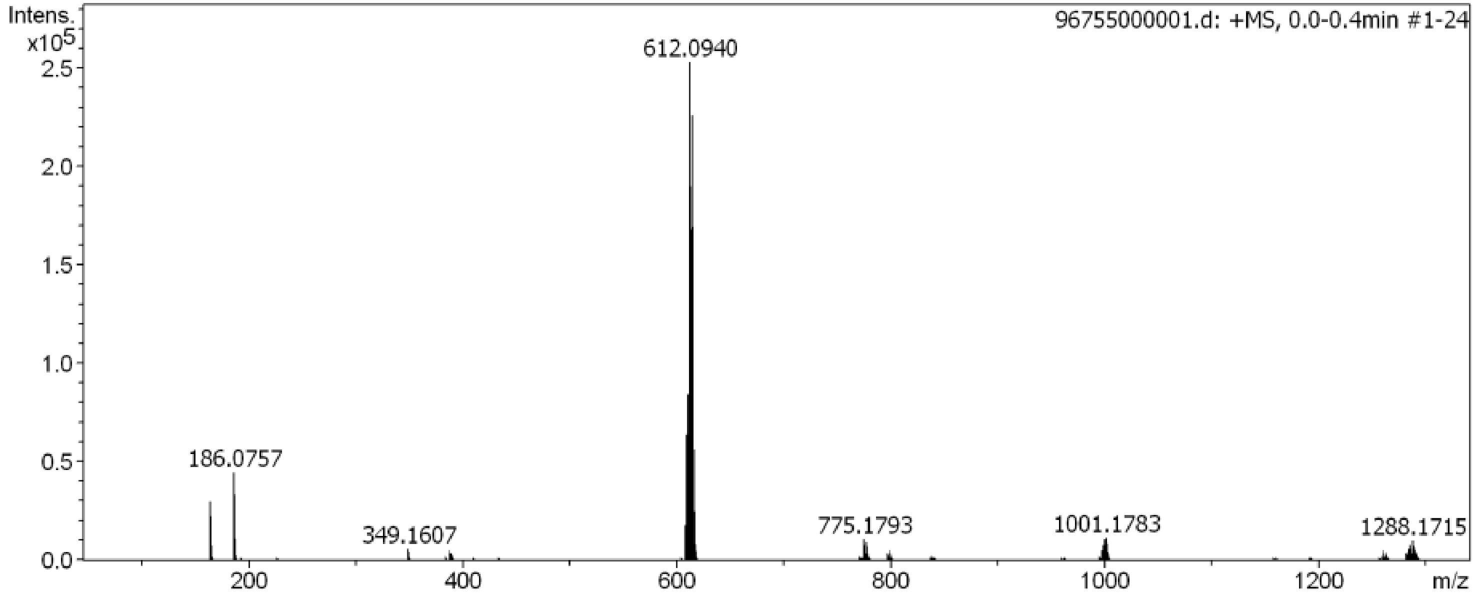

HRMS of 4

ESI (-), MeCN/MeOH + 1% H_2O

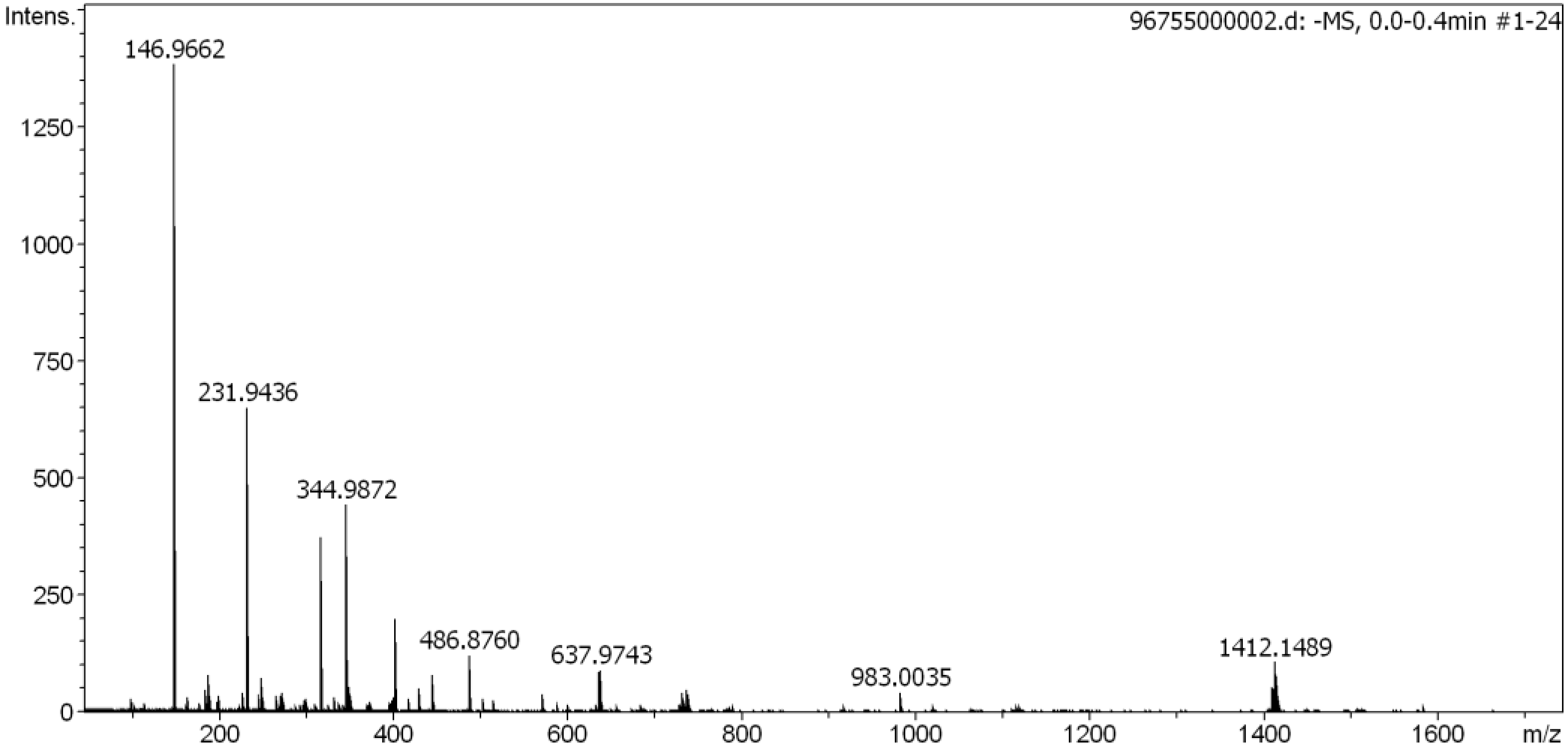

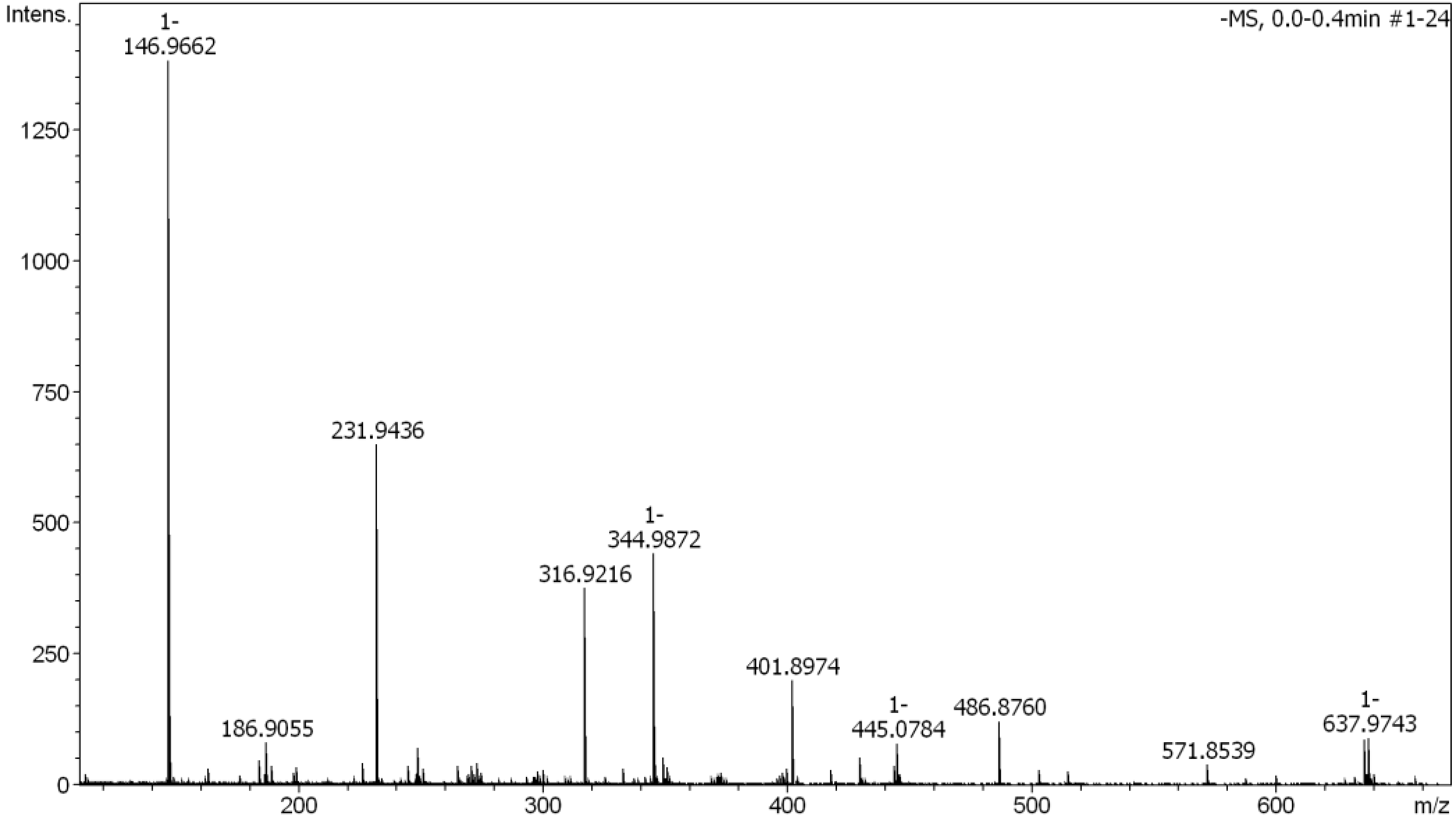

Intens.
-MS, 0.0-0.4min #1-24
1-
146.9662
231.9436
1-
344.9872
316.9216
401.8974
186.9055
1-
445.0784
486.8760
571.8539
1-
637.9743
0
250
500
750
1000
1250
200
300
400
500
600
m/z
